# Prediction and Experimental Validation of Novel STAT3 Target Genes in Human Cancer Cells

**DOI:** 10.1371/journal.pone.0006911

**Published:** 2009-09-04

**Authors:** Young Min Oh, Jong Kyoung Kim, Yongwook Choi, Seungjin Choi, Joo-Yeon Yoo

**Affiliations:** 1 Department of Life Sciences, Pohang University of Science and Technology, Pohang, Republic of Korea; 2 Department of Computer Science, Pohang University of Science and Technology, Pohang, Republic of Korea; University of Pennsylvania School of Medicine, United States of America

## Abstract

The comprehensive identification of functional transcription factor binding sites (TFBSs) is an important step in understanding complex transcriptional regulatory networks. This study presents a motif-based comparative approach, STAT-Finder, for identifying functional DNA binding sites of STAT3 transcription factor. STAT-Finder combines STAT-Scanner, which was designed to predict functional STAT TFBSs with improved sensitivity, and a motif-based alignment to minimize false positive prediction rates. Using two reference sets containing promoter sequences of known STAT3 target genes, STAT-Finder identified functional STAT3 TFBSs with enhanced prediction efficiency and sensitivity relative to other conventional TFBS prediction tools. In addition, STAT-Finder identified novel STAT3 target genes among a group of genes that are over-expressed in human cancer cells. The binding of STAT3 to the predicted TFBSs was also experimentally confirmed through chromatin immunoprecipitation. Our proposed method provides a systematic approach to the prediction of functional TFBSs that can be applied to other TFs.

## Introduction

The ability of any biological system to properly respond to stimuli heavily depends on biochemical cascades of signaling pathways that culminate in the activation of transcription factors (TFs) and the subsequent alteration of gene expression patterns [1]. Information about which genes need to be expressed in a specific cell type at any given time is believed to be encoded in the genome. The molecular machinery used to interpret such genetic information has evolved to ensure the accuracy and specificity of gene regulation. Transcription is a multi-step process requiring the concerted action of many proteins. Transcriptional activators and repressors bind in a sequence-specific manner to promoters or enhancers of target genes. They govern the recruitment of trans-activators, chromatin modifiers, and general transcription factors, including RNA polymerase II, to regulate gene expression [2], [3].

Whole genome approaches to measure genome-wide expression patterns have divulged groups of genes that are co-regulated to exert spatially and temporally controlled cellular responses [4]. Identifying the responsible regulatory modules that govern the coordinated actions of combinatorial transcription factors is crucial for understanding the regulatory circuits of biological processes [5]. For this purpose, computational tools have been developed to aid in the identification of transcription factor binding sites (TFBSs) in the promoters of the co-regulated genes [6], [7], [8]. These computational approaches can be divided into two classes: (1) pattern detection and (2) pattern matching. Pattern detection, also known as de novo motif discovery, finds putative binding sites for unknown TFs that are over-represented in the promoters of co-regulated genes. If the binding specificity of a TF is already known, pattern matching methods are preferred [9]. In the pattern matching approach, DNA sequence information of TFBSs is expressed as a position weight matrix (PWM), which can be used to score potential regulatory sites within a statistical framework [10]. However, because DNA binding sites for TFs are generally short and degenerate, this method is prone to high false positive prediction rates [11].

Based on the observation that conserved non-coding DNA sequences are often important for the regulation of biological functions, cross-species sequence comparisons have been actively integrated to distinguish functional and non-functional TFBSs [12], [13], [14]. The act of incorporating the evolutionarily conserved sequence information in the regulatory regions filters out the non-conserved TFBSs, thereby greatly reduce the false positive prediction rate [15], [16], [17], [18], [19]. Although this approach has been successfully applied to increase the predictive power of motif finding, it is highly sensitive to the algorithm used for sequence alignment and the accuracy of annotated transcriptional start site (TSS) information. Therefore, it has been reported that sequence-based promoter alignments often fail to detect short or degenerate regulatory elements, when evolutionary divergent promoter sequences are aligned [12], [17]. To overcome these limitations, an alignment-free algorithm based on network-level conservation has also been suggested [20].

Signal transducer and activator of transcription 3 (STAT3) belongs to the STAT family of transcription factors, which is activated by Interleukin-6 (IL-6) and related cytokines, such as IL-10, Oncostatin M (OSM), and leukemia inhibitory factor (LIF) [21]. Thus far, seven mammalian STATs (1, 2, 3, 4, 5a, 5b, and 6) have been identified. They all possess a DNA binding domain, an SH2 domain for dimerization, and a C-terminal trans-activation domain [22]. Upon stimulation with extracellular ligand, activated STAT3 forms homodimers or heterodimers with another STAT family member, STAT1, then translocates into the nucleus and binds to cognate regulatory elements in the promoters of STAT-responsive genes. Accumulating evidences suggest that STAT3 also associates with other transcription factors to form enhanceosome complexes in the promoter regions of target genes and controls cooperative gene induction [23], [24], [25]. STAT3 is involved in diverse cellular responses, including cellular differentiation, survival, stem cell renewal, wound healing and systemic inflammation; this has been proven by the phenotypes of genetically modified STAT3 mutant mice [22], [26], [27], [28], [29]. It has been found that STAT3 participates in carcinogenesis, and that the ectopic expression of a constitutively active form of STAT3 (STAT3-C) induces tumor formation in nude mice [30]. Furthermore, the expression of constitutively-active STAT3 has been observed in various types of human cancer including multiple myeloma, colon, ovary, liver, lung, head, and neck cancers [31]. While the regulatory and general trans-activation mechanisms of STAT3 have been thoroughly studied, not too much effort has been made towards the identification of direct target genes of STAT3. The identification of those target genes is crucial for mediating the diverse biological effects of STAT3 signaling.

To characterize STAT3-mediated transcriptional programs, we have developed a computational framework designed to predict STAT3 TFBSs with improved sensitivity and low false positive rate. Through the integration of the microarray data obtained from the STAT3 activation condition and the TFBS prediction tools, we attempted to identify novel STAT3 target genes. Using our STAT-Finder program, we identified eight novel STAT3 target genes among a group of genes that are highly expressed in cancer cells. These were then confirmed through chromatin immunoprecipitation.

## Results

### Overview of STAT-Finder

**Figure 1:**
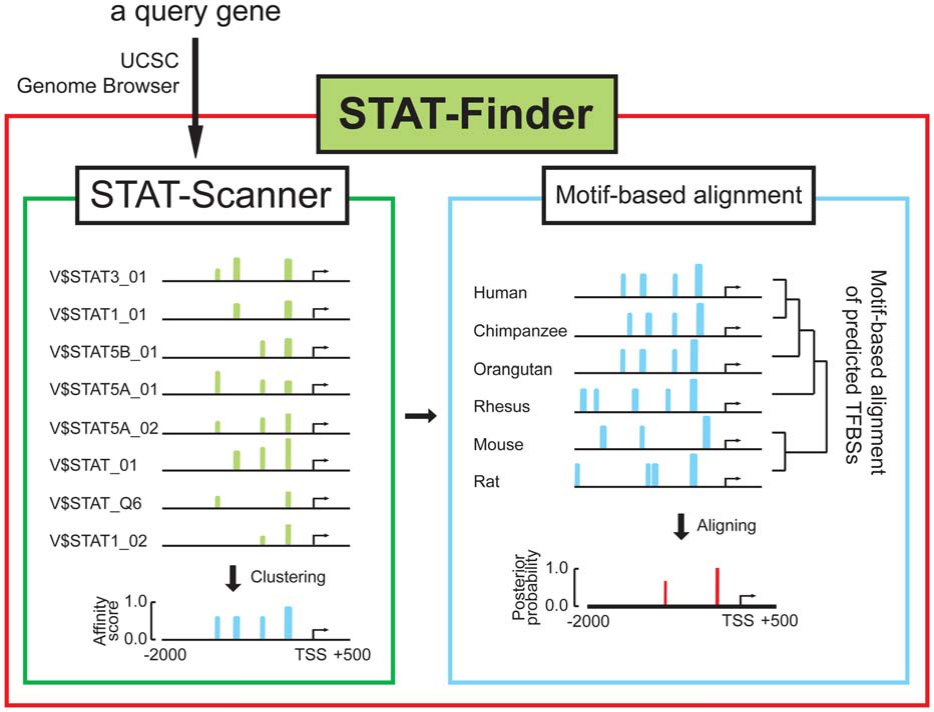
An overview of STAT-Finder. STAT-Finder has two components: The first module, STAT-Scanner, takes a set of six orthologous mammalian promoter sequences as input. Each promoter sequence is searched to mark putative TFBSs using the modified 8 STAT-related PWMs. Binding affinity scores of predicted TFBSs are calculated based on the *P*-values, and a sequence of affinity scores is generated for each promoter. The second module progressively aligns the score sequences and calculates posterior probability to evaluate the degree of motif conservation.

To identify direct STAT3 target genes, we developed a computational framework that predicts functional TFBSs of STAT3 with increased sensitivity and low false positive rate. Our framework, STAT-Finder, was constructed based on two computational components, a TFBS scanning program (STAT-Scanner) and a motif-based alignment program (Figure 1). STAT-Scanner was designed to increase the sensitivity for detecting functional STAT3 TFBSs. A currently available STAT3-specific PWM of TRANSFAC database [32], V$STAT3_01, frequently fails to detect experimentally proven STAT3 binding sites (data not shown). For improved predictive power, STAT-Scanner was therefore designed to use combined PWMs of binding specificity similar to STAT3. Although STAT family members have different physiologic functions and regulate distinct sets of target genes, the targets of individual STAT proteins sometimes overlap, and DNA sequences recognized by STAT family members are similar [21], [22], [23].

For unbiased identification of the PWMs that share sequence similarity with the STAT3-specific PWM, V$STAT3_01, a total of 565 PWMs derived from vertebrate TRANSFAC database [32] were clustered based on their motif similarity (Figure S1). The motif similarity was defined as the *P*-value of the gapped alignment between the two PWMs based on the Kullback-Leibler divergence [33] (See Methods). Total numbers of PWM clusters increased with stringent *P*-value cut-off, reaching maximum cluster numbers of around 10^−16^ *P*-value (Figure S1A). With the *P*-value cut-off of 10^−7^, PWMs assigned for the STAT family members were found in the same cluster. It is noteworthy that PWM clustering did not reveal any non-STAT PWMs that were similar enough to include nor were there any STAT PWMs that were distinctly different (Figure S1B). We chose among them eight PWMs from the STAT family members with high PWM quality scores (>0.6), where each quality score was calculated using the method proposed by Rahmann et al. [34]. The relevance of the selected PWMs for detecting known STAT3 TFBS has been evaluated in the previously identified STAT3 target genes [35] (Figure S2).

To minimize false positive predictions, results from STAT-Scanner were then analyzed using the comparative motif-based alignment tool (Figure 1). This method finds conserved binding sites within the orthologous promoters of six mammalian species by comparing multiple sequences. Within a probabilistic framework, STAT-Finder then evaluates the posterior probabilities of TFBSs as predicted by STAT-Scanner by assigning higher prior probabilities on conserved sites over non-conserved ones.

### Validation of STAT-Scanner

**Figure 2:**
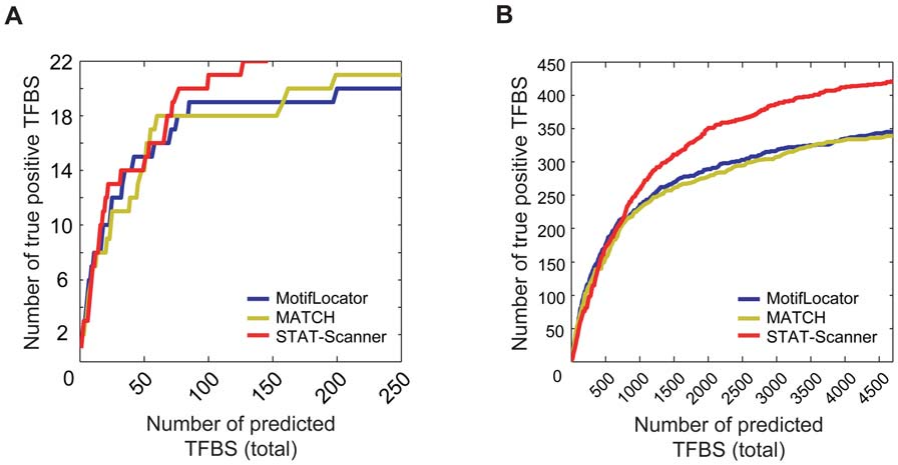
Performance comparison of the STAT3 TFBS prediction tools. Curves for the changes of the number of true positive TFBSs detected using MotifLocator (V$STAT3_01), MATCH (V$STAT3_01), or STAT-Scanner, as a function of total number of predicted TFBSs (A) in the reference set of 22 STAT3 target genes (Table S1) and (B) in the genome-wide STAT3 ChIP-Seq dataset [39].

We first compared the performance of STAT-Scanner with the most practical TFBS prediction tools, MATCH 2.7 [36] and MotifLocator [37]. For this purpose, we collected positive genes with experimentally proven STAT3 binding sites in their promoter regions through literature mining and TRED search (http://rulai.cshl.edu/TRED) [38]. Resulting information on the 22 reference sequences are listed in Table S1. Genomic DNA sequences spanning from 2,000 bp up-stream to 500 bp down-stream of the annotated TSS of each gene were used as input promoter sequences. Prediction of the true positive TFBSs was then plotted as a function of the total predicted TFBS count for different cut-off values. As shown in Figure 2A, STAT-Scanner, which uses combined STAT3-related PWMs, outperforms MATCH and MotifLocator, both of which use the representative STAT3 PWM (V$STAT3_01). We believe the enhanced predictive power of STAT-Scanner was partly due to the usage of combined STAT3-related PWMs, especially since the predictive power of MotifLocator also increased when combined PWMs were used (Figure S3).

We also evaluated the performance of STAT-Scanner using genome-wide STAT3 binding data obtained using embryonic stem cells [39]. Among the 461 genes with STAT3 binding peaks in the 2.5 kb promoter regions, 412 have been accurately predicted by STAT-Scanner to have at least one STAT3 TFBS (Figure 2B). The overall performance of STAT-Scanner was better than those of both MATCH and MotifLocator, as the detection of the same number of true binding sites was achieved by both with significantly lower total numbers of predicted sites. Although MATCH and MotifLocator performed similarly to STAT-Scanner in detecting about 50% of true STAT3 TFBSs, the latter outperforms both by accurately predicting the remaining true sites. We believe this is partly due to the usage of combined STAT-related PWMs which has the capability to enhance the performance of MotifLocator, albeit less than the enhancement for STAT-Scanner, with combined data derived from multiple PWMs (Figure S4). The relative performance of both methods is low compared to that of STAT-Scanner; this can be explained by the fact that their scores on the predicted sites are not directly comparable among different PWMs, thus showing the importance of our scoring scheme in integrating matches to different PWMs. These results also indicate that overlapping PWMs with similar binding specificity are critical to the development of improved strategies to detect functional TFBSs of STAT3 with high predictive accuracy.

### Features of the functional STAT3 TFBS

**Figure 3:**
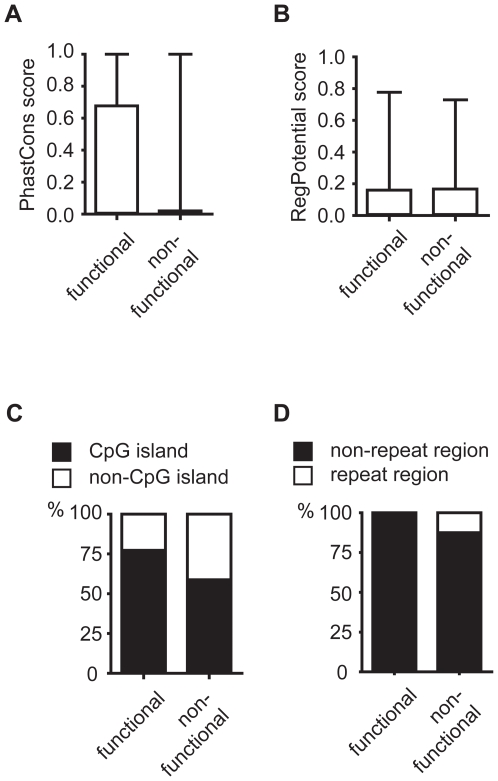
Score distribution of the functional vs. non-functional STAT3 TFBSs as predicted by STAT-Scanner. (A) PhastCons score, (B) Regulatory Potential score, (C) Percentage in the CpG island, and (D) Percentage in the Repeat region.

The ultimate goal of computational prediction is to detect functional TFBSs with a high degree of confidence. To filter out the false positive TFBSs with high affinity scores, we examined various functional constraints such as evolutionary conservation and genome structure of predicted STAT3 TFBS regions. Sequence conservation among multiple species has been proven to constrain functional TFBS [16], [17], [40]. Therefore, we first evaluated the distribution of multispecies conservation scores (PhastCons score) [41] and regulatory potentials (RegPotential score) [42] for positions in the functional and non-functional STAT3 TFBSs detected by STAT-Scanner using the reference set of 22 genes (Table S1). For convenience, we considered a TFBS functional if it was supported by experimental STAT3 binding data; otherwise, the TFBS was considered non-functional. The distribution of PhastCons scores for the non-functional STAT3 TFBSs were skewed towards zero, while PhastCons scores for about 50% of the functional STAT3 TFBS exceeded 0.1 (Figure 3A). In contrast, the distribution of RegPotential scores, which measure the similarity of patterns to those in the known regulatory elements, was similar for positions of the functional and non-functional STAT3 TFBSs (Figure 3B). Next, we investigated the methylation-resistant CpG island features of the STAT3 TFBS-containing regions. Over-representation of the binding sequences for specific transcription factors, such as zinc-finger proteins, in CpG islands has been previously reported [43]. Most of the predicted STAT3 TFBSs are located inside CpG islands [44], but the genomic distribution is not significantly altered among the functional and non-functional STAT3 TFBSs (Figure 3C). Repeat elements [45] in the genomic sequence might compromise the functions of transcription factors, as none of the functional STAT3 TFBSs have been identified inside the repeated regions (Figure 3D). In summary, motif conservation, a major constraint that distinguishes between functional and non-functional STAT3 TFBSs, has therefore been included in STAT-Finder.

### Validation of STAT-Finder

**Figure 4:**
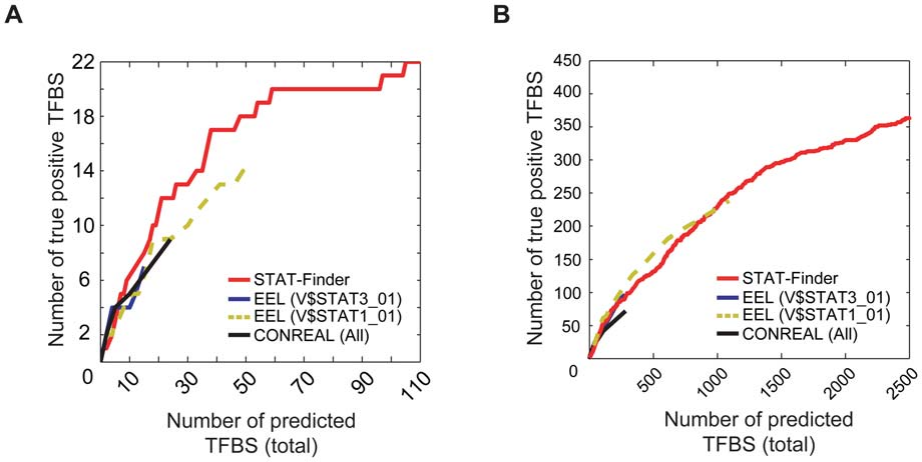
Performance comparison of the comparative alignment tools. Curves for the changes of the number of true binding sites detected using EEL (V$STAT3_01 or V$STAT1_01), CONREAL (All; combined PWMs of V$STAT3_01 and V$STAT1_01), or STAT-Finder, as a function of total number of predicted TFBSs (A) in the reference set of 22 genes (Table S1) and (B) in the genome-wide STAT3 ChIP-Seq dataset [39].

We next evaluated the performance of STAT-Finder compared to other comparative methods, namely, EEL [46] and CONREAL [12]. Given that EEL performs pair-wise alignment based on the matches to a single PWM, we compared the performance of EEL using each PWM (V$STAT3_01 and V$STAT1_01) separately. Meanwhile, the performance of CONREAL was examined by combining both PWMs. We tested the prediction accuracy of STAT-Finder in the two positive data sets with STAT3 bindings. STAT-Finder exhibited better performance compared to EEL using V$STAT3_01, EEL using V$STAT1_01, or compared to CONREAL in predicting true STAT3 TFBSs in the 22 previously identified positive genes (Figure 4A). Note that both EEL and CONREAL failed to detect about 40–60% of true positive STAT3 sites even at the minimum cut-off value, while STAT-Finder found all of these. These data indicate that STAT-Finder showed better performance in terms of finding true positive STAT3 TFBSs that the other comparative programs missed. It was made more evident when we searched STAT3 TFBSs using EEL or CONREAL in the data sets with genome-wide STAT3 binding. Although the overall performance of the STAT-Finder was similar to EEL in detecting 56% of true STAT3 TFBSs, only STAT-Finder was capable of detecting the remaining 30% of the true sites (Figure 4B). Our data suggest that the improved sensitivity of STAT-Finder could be attributed to the usage of combined STAT-related PWMs, which evidently overcame the performance limitations of V$STAT3_01.

We next attempted genome-wide prediction of STAT3 binding in the human promoter regions. For this purpose, we first estimated the cut-off value of the motif conservation score (MCS) to identify conserved functional STAT3 TFBSs. The degree of conservation of the predicted TFBS, which was determined by calculating MCS, was integrated with the affinity scores by STAT-Scanner (See Methods). The confidence score at each MCS was evaluated using the 2.5 kb promoter sequences of all annotated human genes and orthologous mouse genes. The confidence score determines the probability that a given TFBS is not conserved by chance. As cut-off values of MCS increased, the total number of predicted STAT3 TFBSs decreased at a slower rate than the average number of aligned instances of control motifs, resulting in escalated confidence scores at MCS values higher than 0.9 (Figure S5). Using STAT-Finder, we performed a genome-wide search for STAT3 TFBSs in the human promoter regions. Among the 15461 human genes with identified orthologs in the mouse, about 7600 genes were predicted to have putative STAT3 binding sites within the 2.5 kb promoter region, at the probability threshold of 0.9. Significant enrichment of STAT3 TFBSs could be predicted at the proximal upstream regions of TSS using STAT-Scanner and STAT-Finder [35], [39] (Figure S6).

### Identification of novel STAT3 target genes in the cancer cells

Constitutive activation of STAT3 and over-expression of its target gene have been suggested to play critical roles in human carcinogenesis [12], [31], [47], [48], [49], [50]. To determine whether or not STAT-Finder is useful in identifying novel STAT3 target genes, we applied this program to a group of genes that are over-expressed in human cancer cells. We integrated microarray data obtained from the expression module map of genes up-regulated in cancer [51] and data derived from the A549 cells over-expressing a constitutively active form of STAT3 [52].

**Figure 5:**
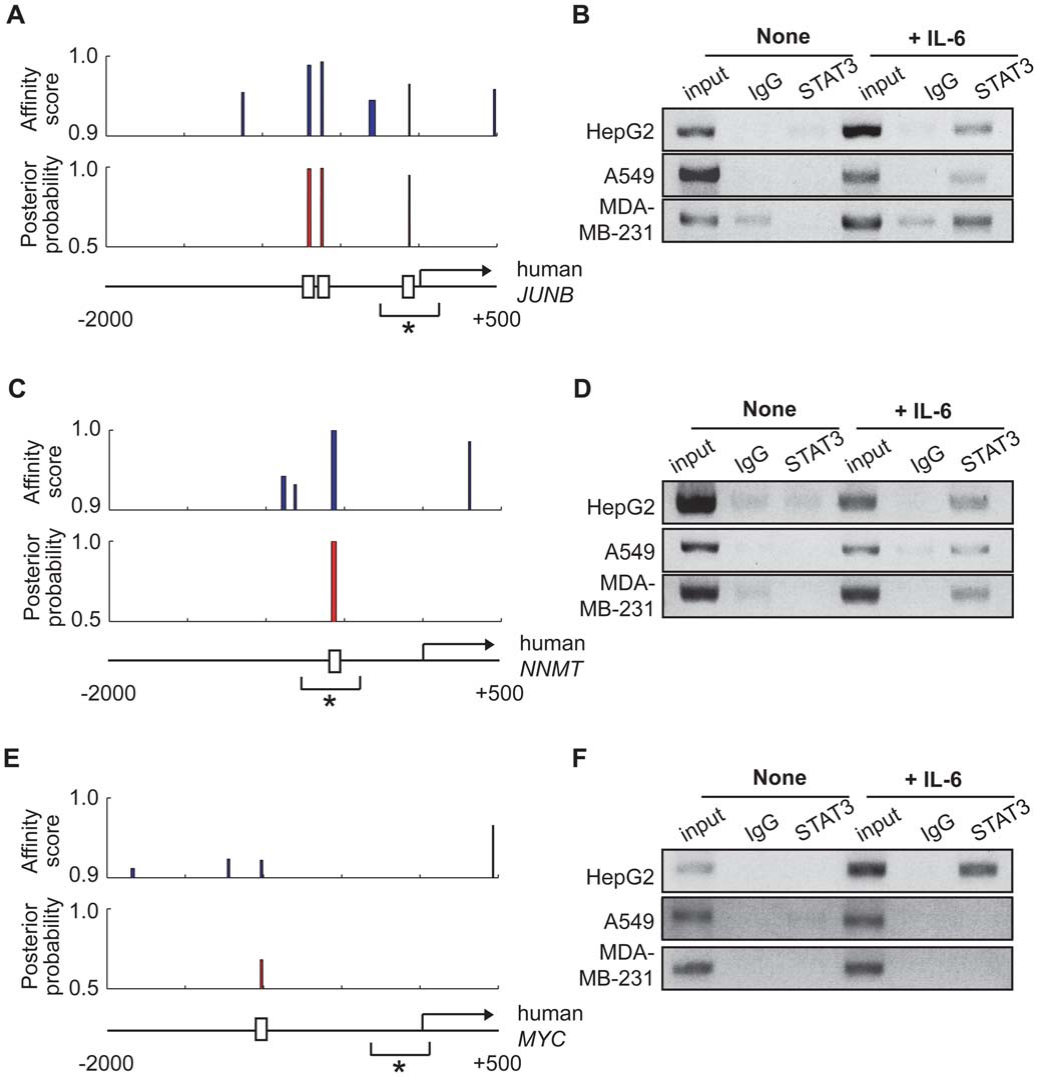
Experimental validation of STAT3 binding to the known STAT3 TFBSs. (A,C,E) The affinity score from STAT-Scanner (top) and the posterior probability from STAT-Finder (middle) of predicted STAT3 are plotted in the sliding windows for a 2.5-kb promoter region across the *JUNB* (A), *NNMT* (C), and *MYC* (E) genomic loci. The open square at bottom indicates the predicted TFBS with the posterior probability higher than 0.95; while the asterisk (*) in the promoter region depicts the known STAT3 TFBS. (B, D, F) Chromatin immunoprecipitation analysis with an anti-STAT3 antibody: Reported STAT3 TFBSs of *JUNB* (B), *NNMT* (D), and *MYC* (F) were PCR amplified using the primers specific binding sites (*) from the input and immunoprecipitated cell lysates, derived from the non-stimulated or IL-6 (10 ng/ml) + IL-6sR (10 ng/ml)-stimulated HepG2, A549, and MDA-MB-231 cells.

Among the 33 genes that are commonly up-regulated, eleven have already been reported to be regulated by STAT3 (Table 1). Using this group of genes, we examined whether or not STAT-Finder could detect experimentally proven STAT3 TFBSs. It is noteworthy that we were able to analyze only a fraction of the promoter sequences, mainly due to alternative promoter usage and the poorly annotated TSS information available. STAT-Finder detected three putative STAT3 binding sites in the *JUNB* promoter region including one site that has previously been reported to be a STAT3 binding site [53] (Figure 5A). Using three different cell lines derived from human cancer patients, we confirmed STAT3 binding to the *JUNB* promoter by chromatin immunoprecipitation (Figure 5B). STAT-Finder also successfully detected one STAT3 TFBS in the Nicotinamide N-methyltransferase (*NNMT*) promoter region, a recently identified STAT3 target gene [54] (Figure 5C, D). Interestingly, STAT-Finder was unable to detect known STAT3 TFBS in the *MYC* promoter region (Figure 5E), even though *MYC* has been reported to be a STAT3 target [55]. It has also been reported that STAT3 binding to the promoter region of the *MYC* gene requires a site that is different from the consensus STAT3 binding sequences, but is similar to E2F TFBS, indicating that, in this case, STAT3 binding depends on the presence of other transcription factors [55]. Using primer sets that detect known STAT3 binding sites in the *MYC* promoter, we were able to confirm its binding upon IL-6 stimulation in HepG2 cells (Figure 5F). These results suggest that STAT-Finder could efficiently detect binding sites for STAT3 only if their binding does not depend on the presence of other *cis* or *trans* factors.

**Table 1:** Putative STAT3 target genes.

Gene	Entrez ID	aFold Change (log_2_)	aFDR	bCancer Module #	bCancer Module (*P*-value)	Reported STAT3 regulation	Reported STAT TFBS	^c^Reference	Remark for experiment
*AKAP12*	9590	3.955	0	3	<1e-14	-	-	-	Putative target
*ATF3*	467	5.885	0	197	0.002153	-	-	-	Putative target
*CCL2*	6347	7.205	0	3	<1e-14	+	+	[73]	
*CITED2*	10370	2.911	0	3	<1e-14	+	+	[74]	
*CXCL2*	2920	2.092	0	197	0.021719	+	-	[75]	
*DDEF2*	8853	2.184	0	98	1.05E-12	+	-	[76]	
*DUSP5*	1847	2.782	0	98	0.001713	-	-	-	Putative target
*ETS2*	2114	3.039	0	197	0.000899	-	-	-	
*FOSL1*	8061	4.032	0	98	7.13E-05	-	-	-	
*HIC2*	23119	2.817	0	17	0.007665	-	-	-	Putative target
*JUN*	3725	3.194	0	197	5.36E-05	+	-	[77]	
*JUNB*	3726	2.436	0	17	1.07E-05	+	+	[53]	Positive control
*LDLR*	3949	3.548	0	3	<1e-14	-	-	-	
*LOXL2*	4017	2.139	0	3	<1e-14	-	-	-	
*MAFF*	23764	5.273	0	3	<1e-14	+	+	[78]	
*MAP2K3*	5606	2.739	0	18	0.001053	-	-	-	
*MYC*	4609	2.053	0	126	<1e-14	+	+	[55]	Positive control
*NNMT*	4837	2.041	0	3	<1e-14	+	+	[54]	Positive control
*NP*	4860	2.043	0	3	<1e-14	-	-	-	Putative target
*NPC1*	4864	5.916	0	18	1.1E-06	-	-	-	
*PLAUR*	5329	2.89	0	3	2.53E-12	-	-	-	
*PLEC1*	5339	2.982	0	18	0.016729	-	-	-	
*PLEKHC1*	10979	2.188	0	3	0.045975	-	-	-	
*PMAIP1*	5366	2.031	0	54	1.7E-05	+	-	[79]	
*PXN*	5829	2.217	0.098	18	5.01E-06	-	-	-	
*SERPINE1*	5054	2.312	0	3	<1e-14	-	-	-	Putative target
*SGK*	6446	2.826	0	3	0.004895	+	+	[80]	
*SLC2A3*	6515	6.191	0	17	2E-06	-	-	-	Putative target
*TAF1A*	9015	2.661	0.042	124	0.005465	-	-	-	
*THBS1*	7057	3.25	0	3	<1e-14	-	-	-	Putative target
*UGCG*	7357	6.265	0	3	2.21E-08	-	-	-	
*WEE1*	7465	2.172	0.069	57	<1e-14	-	-	-	
*ZYX*	7791	2.124	0	3	<1e-14	-	-	-	

a Analyzed microarray data of A549 cell line over expressing STAT3C [52] using SBEAMS [72].

b Analyzed data of the cluster in the Cancer Module Map [51] (http://robotics.stanford.edu/~erans/cancer/).

**Figure 6:**
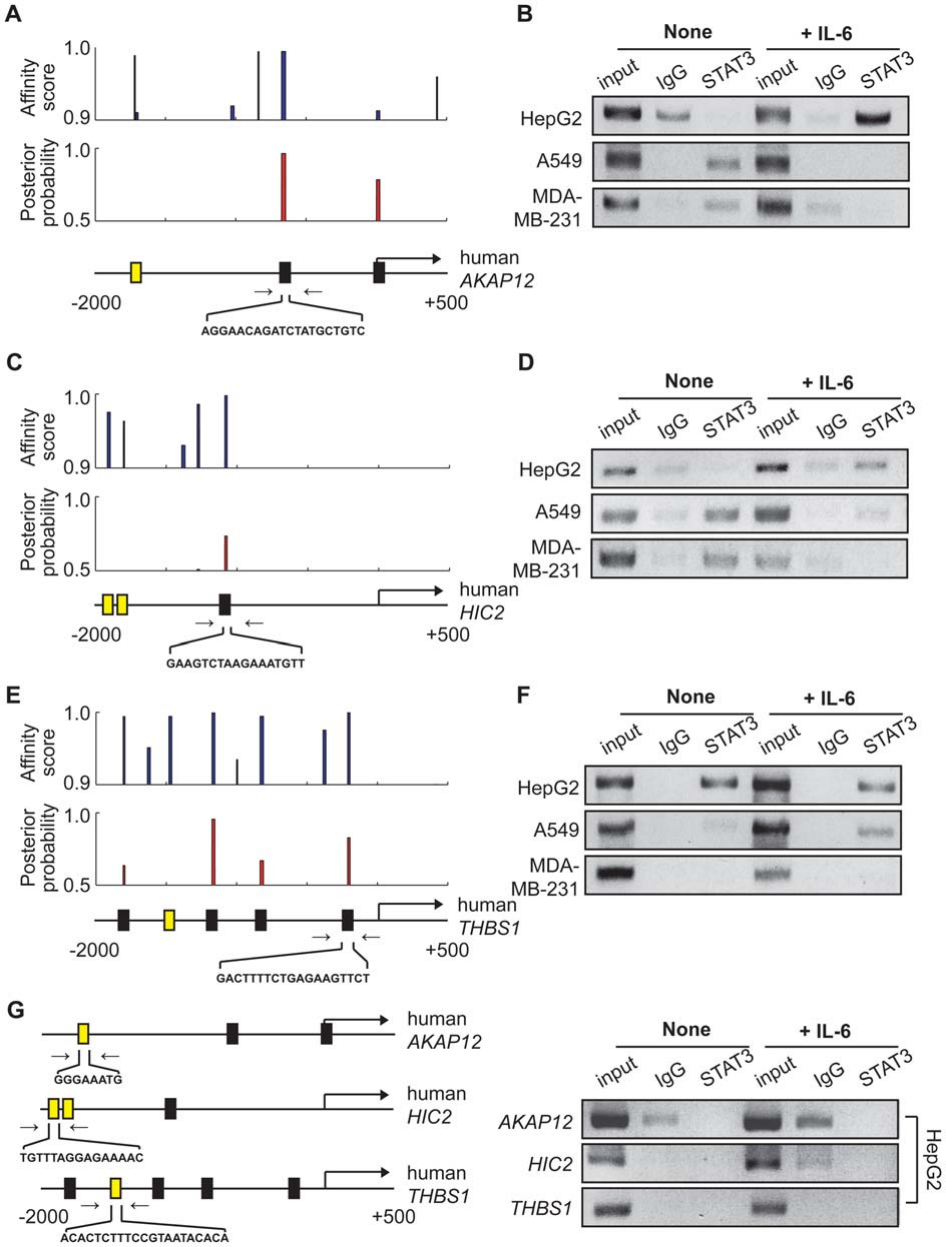
Experimental validation of STAT3 binding to the novel STAT3 TFBSs. (A, C, E) The affinity score (top, STAT-Scanner) and posterior probability (middle, STAT-Finder) of predicted STAT3 TFBSs are plotted in the sliding windows for a 2.5-kb promoter region across the *AKAP12* (A), *HIC2* (C), and *THBS1* (E) genomic locus. The closed square at the bottom indicates the predicted TFBS with posterior probability >0.5; while the yellow square shows the predicted TFBS with no conservation. (B, D, F) ChIP analysis with an anti-STAT3 antibody. Putative STAT3 TFBSs of the *AKAP12* (B), *HIC2* (D), and *THBS1* were PCR amplified using the primer sets indicated by inverse arrows. (G) ChIP analysis with an anti-STAT3 antibody. Predicted TFBSs with no conservation in the human *AKAP12*, *HIC2*, and *THBS1* genes were PCR amplified using the primer sets indicated by inverse arrows.

We next examined whether or not we can identify novel target genes of STAT3 using STAT-Finder. For this purpose, we selected genes with conserved TSS (Table 1) and determined the presence of putative STAT3 TFBSs using STAT-Finder in their promoter regions. STAT-Finder successfully detected putative STAT3 TFBSs with high probabilities in the promoter regions of *AKAP12* (A-kinase anchoring protein 12), *HIC2* (hyper-methylated in cancer 2), and *THBS1* (Thrombospondin 1). STAT3 binding to these predicted sites was experimentally confirmed by ChIP assay (Figure 6A–F). To verify the specificity of STAT-Finder, we also assayed the binding of STAT3 to the sites that were not conserved, but were present in the promoters of human orthologous genes. In contrast to the conserved STAT3 TFBSs, we could not detect STAT3 binding to the non-conserved STAT3 TFBSs in human cancer cell lines (Figure 6G). STAT3 binding to other predicted STAT3 TFBSs present in the promoter regions of *ATF3* (activating transcription factor 3), *DUSP5* (dual specificity phosphatase 5), *SERPINE1* (serpin peptidase inhibitor, class E), *NP* (nucleoside phosphorylase), and *SLC2A3* (solute carrier family 2, facilitated glucose transporter, member 3) were also experimentally validated (Figure S7). Finally, we studied whether or not other computation tools such as EEL or CONREAL could also accurately detect STAT3 target sites that have been identified and validated in this study. Of 10 promoter sequences containing experimentally proven 10 STAT3 binding sites (Figure 5, 6 and S7), STAT-Finder predicted a total of 29 STAT3 binding sites including all of the 10 experimentally validated STAT3 binding sites. Meanwhile, EEL and CONREAL detected only 5 (50%) and 2 (20%) validated STAT3 binding sites among 23 and 6 total predictions, respectively, thereby indicating that STAT-Finder has better performance in terms of identifying novel target genes of STAT3 (Figure S8).

## Discussion

We presented a computational framework for identifying functional STAT3 TFBSs in mammalian promoters. The first compartment, STAT-Scanner, was designed to predict functional STAT3 TFBSs with improved sensitivity. By using comparative motif-based alignments, STAT-Scanner was linked to STAT-Finder to minimize false positive predictions. Our proposed method was tested using previously identified STAT3 target genes and was successfully applied to the identification of novel target genes.

Our strategy in developing STAT-Finder relied on several assumptions. First, the DNA binding specificity of STAT3 is shared by other STAT family members. STAT transcription factors bind to similar DNA sequences, and the similar DNA binding specificity of various STAT transcription factors, such as STAT1, STAT5A/5B, or STAT6, have been experimentally proven [56]. It has also been noted that integration of the overlapping matches detected by matrices from the same family members greatly reduces the number of total predicted TFBSs, and hence decreases the rate of false positive detection [57]. Furthermore, it has been recently reported that roughly half of TFs recognize multiple sequence motifs [58]. Therefore, a conventional motif scanning approach using a single PWM for each TF has an intrinsic limitation in detecting all functional TFBSs. As a result, the predictive power of STAT-Scanner was significantly enhanced by integrating STAT-related PWMs. The second assumption, used in the motif-based alignments, is that the relative locations of functional TFBSs are conserved among closely related mammalian species. In yeast, highly conserved TFBSs for a set of TFs exhibit relatively low spatial deviations (∼150–200 bp) [20]. Likewise, we found that, for six mammalian species, known STAT3 TFBSs are located within a similar spatial distribution on each promoter.

Using STAT-Finder, we have identified a list of STAT3 target genes that are over-expressed in human cancer cells. Likewise, STAT3 binding to the predicted TFBSs has been experimentally verified in IL-6 stimulated human cancer cell lines. Interestingly, STAT3 was recruited to the predicted TFBS in a cell type-specific manner. For example, STAT3 binding to the predicted TFBSs in the promoter regions of the *AKAP12* and *HIC2* genes was observed in un-stimulated but not in IL-6 stimulated A549 and MDA-MB-231 cells. However, in the HepG2 cells, STAT3 was recruited to the same TFBS only after IL-6 stimulation (Figure 6). In contrast, STAT3 binding to the promoter regions of *MYC*, *SERPINE1*, *NP*, and *SLC2A3* was only detectable in IL-6 stimulated HepG2 cells, but not in A549 or MDA-MB-231 cells (Figure 6, Figure S7). Furthermore, it is evident that STAT3 binding to the predicted TFBSs in the promoters of the candidate target genes does not guarantee the expression of that gene. Although the expression of most of the target genes had been altered upon STAT3 binding to the promoter, we found that STAT3 binding to target sites did not always correlate with gene expression in the cell lines tested (Oh, YM, unpublished data). This suggests that STAT3 binding to target sites is not sufficient in inducing gene expression, and tissue-specific transcription factors, or trans-activators that specifying modification in the chromatin region may also be required [59], [60], [61], [62].

A *cis*-regulatory module comprises a cluster of multiple TFBSs that cooperatively-interact with TFs to control gene expression. The identification of *cis*-regulatory modules for specific gene regulation is a challenging step towards understanding genome-wide transcription regulatory networks in mammalian genomes. Therefore, it is necessary to efficiently predict functional TFBSs for individual TFs. We expect that our comparative approach can be applied to other TFs with some restrictions. First, the efficiency of our program depends on the degree of evolutionary conservation among the six mammalian species. Therefore, DNA binding sites for TFs engaged in species-specific gene regulation may not be predicted. It is noteworthy that the frequent gain or loss of TFBSs in the intergenic regions leads to the evolution of transcriptional circuits [63]. Second, our program may not be applied to TFs that rely on other DNA binding proteins for recruitment into DNA. Third, because we only compared 2 kb of upstream promoter sequence relative to the annotated TSS, DNA binding sites of TFs that are enriched in regions distal to the TSS might be overlooked by our program. Although *cis*-regulatory regions that lie >100 kb away from the TSS exist, it has been suggested that most functional TFBSs are highly enriched in regions proximal to the TSS [40], [64]. Another limitation is the amount and quality of annotated TSS information obtained from diverse mammalian species. With the exception of those from humans and mice, annotated TSS information for most of the mammalian genomes is not available, and correct TSS information is crucial for the identification of evolutionarily conserved TFBSs based on motif-based alignments. Obtaining accurate and reliable prediction of functional TFBSs in the promoter region is a critical step in deciphering the regulatory code of the complex transcription regulatory networks that govern diverse biological responses. Given that our proposed method is based on a multiple-motif model, we believe it can be applied to other TFs, with some modifications, and may serve as a basic tool to discover important *cis*-regulatory features.

## Materials and Methods

### Clustering of STAT3-related PWMs

We used dynamic programming to find the optimal gapped alignment between two PWMs. We denote by 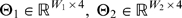 the two PWMs of length 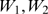 over 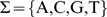 to align, where 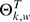 represents row 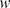, each entry is non-negative and 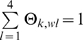. The optimal pair-wise alignment can be found by the following steps. We first construct a matrix 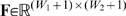 whose 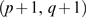-element 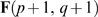 is the score of the optimal alignment between the sub-matrices 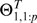 and 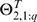. Initially, we set 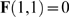, 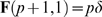 and 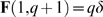 for all 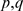, where 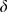 is a gap penalty. We then build up the matrix 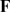 using the following recurrence:
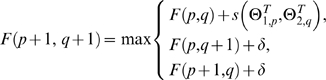
where 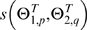 is the match score which is defined by the Kullback-Leibler divergence
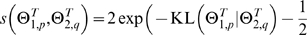
where
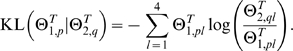



To define the similarity between the two PWMs, we assessed the optimal alignment by calculating the *P*-value. The *P*-value of the observed alignment score was calculated by estimating the score distribution of 1000 randomly-permuted PWMs via the Gaussian distribution. In this study, the gap penalty 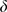 was set to 0.5.

### Prediction of putative STAT TFBSs: STAT-Scanner

We searched putative TFBSs of the STAT family in input promoter sequences and evaluated their binding affinity scores using STAT-Scanner. Given a set of position count matrices obtained from TRANSFAC 9.4 [32], we reconstructed STAT-related PWMs to compute the *P*-values of the match scores using a method for calculating the exact distribution of scores [65], [66]. Briefly, our method consists of three steps. First, we transformed each position count matrix into the corresponding position frequency matrix (PFM) by adding position-dependent pseudo-counts [34]. This position specific regularization leaves the conserved positions of the matrix relatively unchanged. Second, from the regularized PFM, we reconstruct a position weight matrix (PWM) whose element is the log-odds score between the PFM and background model, defined by a zero-order Markov chain. Then, the match score is defined by the sum of the log-odds scores. To account for the effect of the uneven distribution of “GC” and “AT” content, we used six different background models that were constructed based on clusters of nucleotide compositional vectors of the whole mouse promoter sequences available from Ensembl [67]. For clustering, we used the *k*-means clustering algorithm. Finally, to determine statistically significant TFBSs, we calculated the exact distributions of the match scores under the background model assumption. From the distributions, we calculated the type-I sequence error probability *α_n_*(*s*), which measures the probability that at least one site within a sequence of length *n* (*n* = 500 as proposed by [34]) has a match score larger than or equal to *s*, under the assumption that the sequence is generated from the background model. We then converted the match score, *s*, into the affinity score, *t*, defined by 1- *α_n_*(*s*). This conversion makes it easy to define a threshold, γ, of the affinity score within a statistical hypothesis testing framework. In addition, it is also plausible to directly compare the affinity scores of different STAT-related PWMs.

Given a set of STAT-related PWMs and an input promoter sequence, STAT-Scanner first computes the nucleotide composition of the input sequence in order to select the nearest background model, and searches TFBSs whose affinity scores are larger than the threshold, with the PWMs constructed by the chosen background model. We used eight STAT-related PWMs, V$STAT_01, V$STAT1_01, V$STAT3_01, V$STAT5A_01, V$STAT5B_01, V$STAT5A_02, V$STAT1_02, and V$STAT_Q6, and combined all overlapping sites of the eight PWMs into one with the maximum affinity score. Notably, the max operator is applicable because the affinity scores of different PWMs are directly comparable.

### Prediction of the conserved STAT TFBSs: STAT-Finder

STAT-Finder was designed to minimize the false positive discovery of predicted TFBSs using comparative sequence comparisons. It searches conserved sites within the promoters of six orthologous species by sequences of affinity scores. To diminish probabilities of misalignments, we used score sequences defined by STAT-Scanner as a first approximation of the conserved regulatory regions. We regarded a region with nonzero affinity scores within the score sequence as the regulatory region. We focus on the regulatory regions of multiple alignments by ignoring the non-conserved regions. We progressively aligned the six score sequences obtained from orthologous promoter sequences, according to the phylogenetic tree of all six mammalian species, and evaluated the degree of conservation by calculating the Motif Conservation Score (MCS). The MCS value of an aligned TFBS ranges from 0 (non-conserved) to 1 (most conserved).

Motif-based alignment tool consists of two main parts to align multiple sequences with different affinity scores of STAT TFBSs. The first part is a pair-wise global alignment module that finds an optimal alignment between two sequences. We adapted a variant version of the Needleman-Wunsch algorithm [68], with a modification in the scoring function for the match between two affinity scores. The second part is a progressive alignment module that determines a multiple motif alignment among orthologous promoter sequences derived from six mammalian species. The basic concept of this approach is to sequentially perform pair-wise alignments between two sequences of affinity scores, between a sequence and a profile, or between two profiles, according to the phylogenetic tree of the six species. The profile is a set of aligned sequences with gaps. This progressive alignment efficiently aligns multiple sequences with reasonable accuracy. In the given multiple motif alignment, we computed the motif conservation score (MCS) at each aligned position by taking the average of the aligned affinity scores. The affinity score of the gap was set to zero and the “N” character was not considered when calculating the average.

STAT-Finder has a unique feature that detects not only conserved binding sites but also non-conserved ones with very strong binding signals to rectify the unavoidable alignment error. Before describing our probabilistic model, we first explain our notations to define our model. Among the 8 STAT3-related PWMs, we denote by 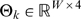 the *k* ^th^ PWM of length 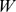 over 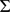, where 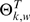 represents row 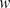, each entry is non-negative and 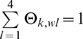. The background model 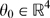, which describes frequencies over the alphabet within non-binding sites, is defined by a zero-order Markov chain. We assume the background model is known in advance and estimated from the whole mouse promoter sequences.

Suppose we have a promoter sequence 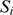 which is a string of length 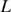 over the alphabet 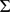. In order to allow for multiple binding sites per sequence, we represent the sequence 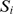 as a set of overlapping subsequences 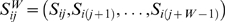 of length 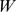 starting at position 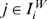, where 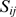 denotes the letter at position 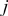 and 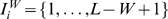. Let us introduce a latent variable matrix 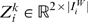 in which the *j* ^th^ column vector 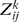 is defined as a 2-dimensional binary random vector 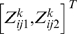 such that 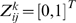 if a binding site of the *k* ^th^ PWM starts at position 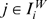. Otherwise, 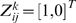.

Our probabilistic model has the following specification for defining the joint distribution. First, latent variables 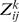 indicating the starting positions of binding sites of the *k* ^th^ PWM are governed by the probability 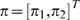 such that 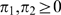 and 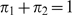. The prior probability of 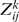 is specified by
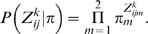



For each latent position 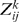, the probability distribution of the subsequence 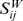 is given by
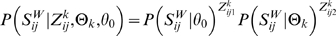
where
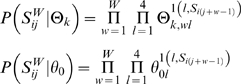
where 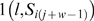 is an indicator function which is 1 if 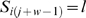, and 0 otherwise.

The objective of probabilistic inference in our model is to calculate the posterior probability 
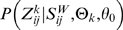
 because this probability evaluates the degree of being a true binding site of the subsequence using our prior knowledge and given data. The posterior probability can be obtained by using Bayes' theorem
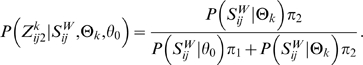



The degree of conservation of each subsequence can be easily incorporated into our probabilistic framework by assigning relatively higher prior probability 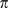 on 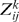 than non-conserved one. In this work, we used the following settings: 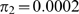 for non-conserved subsequence (the expected number of binding sites is 1 when the promoter sequence is 2500 (double stranded)) and 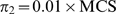. The eight different posterior probabilities of the eight STAT3-related PWMs across the latent positions are integrated by taking the maximum value and the probability cutoff value was set to 0.5.

### Motif-based pair-wise alignment

We denote two promoter sequences of lengths 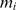 and 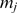 by 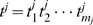 and 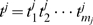, where 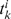 and 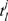 are the affinity scores of STAT3 TFBSs. If the score is smaller than a threshold 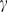, we cut the score to 0. We also set the score to −1 if the corresponding site contains the ambiguous “N” characters. With this setting, the optimal pair-wise alignment between 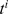 and 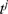 can be found by dynamic programming. We first construct a matrix 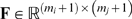 whose 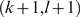-element 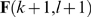 is the score of the optimal alignment between the segments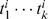 and 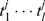. For initialization, we set 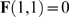,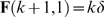 and 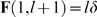 for all 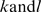, where 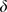 is a gap penalty. We then build up the matrix 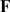 using the following recurrence: 
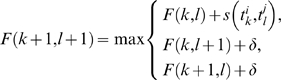
where 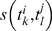 is the sequence match score between 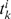 and 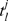 which is defined by
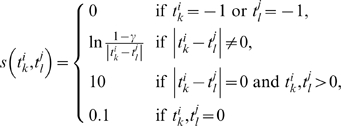



We construct the optimal alignment by tracing back the choices that result in the final value of 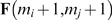.

### Motif-based profile alignment

We denote two profiles of lengths 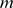 and 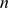 by 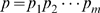 and 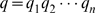, where 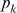 and 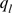
are the aligned affinity scores of STAT3 TFBSs constructed from disjoint promoter sequences indexed by 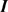 and 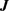, respectively. The pair-wise profile alignment can be also found by dynamic programming. The profile match score 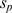 is defined by the average of the sequence match score:
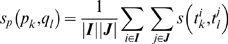
where the sequence match score 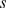 is slightly modified to deal with the gap “-“ by setting 
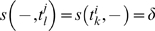
 and 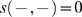. In this study, the score threshold 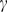 and gap penalty 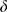 were set to 0.8 and −0.1, respectively.

### Estimation of MCS confidence score

To generate randomly shuffled control motifs, we manually aligned STAT-related PWMs without gaps by looking up the core regions (TTCCNGGAA). We excluded V$STAT5A_02 (homotetramer) because it was not aligned with other PWMs. The operation for random permutation was then applied to the aligned PWMs to generate 100 control motifs. Based on the assumption that the control motifs should have occurrence rates similar to the real motif, we selected 42 control motifs that detect similar numbers of TFBSs in the reference data set (±15%). Among them, we chose 10 motifs that were most dissimilar to V$STAT_01, based on the inter-motif distance measure (cut-off: 0.25) [69]. The confidence level at each MCS was then calculated using the following equation: (the total number of TFBSs of the real motif - the average number of TFBSs of the control motifs)/the total number of TFBSs of the real motif. This value represents the fraction of the number of conserved TFBSs above the ones that occurred by chance.

### Retrieving information for promoter sequence

Human and mouse promoter sequences (−2000∼ +500 bp of the annotated transcription start sites) were downloaded from Table Browser of the UCSC genome browser [70]. We used hg18 and mm9 for human and mouse genome UCSC version, respectively. Orthologous promoter sequences of chimpanzee, orangutan, and rhesus were obtained by blatting the 2.5-kb human promoter sequences into the UCSC genome browser of each species [70]. Rat promoter sequences were obtained by blatting the 2.5-kb mouse promoter sequences into the UCSC genome browser of rat. For each 2.5-kb promoter sequence, PhastCons scores, Regulatory potential scores, CpG island, and regions for repeated elements were also obtained through Table Browser of the UCSC genome browser.

Genome-wide STAT3 ChIP-Seq data set was obtained from [39]. In this data set, we first selected 461 genes with STAT3 binding peaks located in 2.5kb promoter regions, among which 412 genes have at least one site predicted by STAT-Scanner (cut-off: 0.2) that is overlapped with experimentally identified regions (within 150 bp of STAT3 binding peaks). We next defined true positive sites as those that are overlapped with the STAT3 binding regions and that match to the highest scoring site predicted by STAT-Scanner, as suggested by [71].

### Microarray data analysis

Cancer module map information was downloaded from the web browser (http://ai.stanford.edu/~erans/cancer/). We used Cancer Module 3, 17, 18, 54, 57, 98, 124, 126, and 197, which contain commonly up-regulated genes across liver cancer, B lymphoma, grade3 breast cancer, and stimulated macrophages. Microarray CEL data files of STAT3-C over-expressed cells were obtained from Dr. E.B. Haura (University of South Florida, Tampa) [52]. Microarray data was analyzed using the SBEAMS program [72]. The data set was normalized by the global quantile scaling method (GC-RMA) and filtered to include differentially expressed genes with more than two fold change, with FDR <0.1 and *P*-value <0.01 (*t*-test).

### Cell culture

Human hepatocarcinoma cell line, HepG2, was maintained in MEM supplemented with 10% FBS (Hyclone, Logan, UT) and 1% penicillin/streptomycin (Invitrogen, Carlsbad, CA). Human lung carcinoma cell line, A549, and breast cancer cell line, MDA-MB-231, were cultured in DMEM with 10% FBS and 1% penicillin/streptomycin. For IL-6 stimulation, cells were treated with rhIL-6 (10 ng/ml) and rhIL-6sR (10 ng/ml) (R&D Systems, Minneapolis, MN) for 15 minutes.

### Chromatin Immunoprecipitation (ChIP)

ChIP assays were performed as described with minor changes [64]. Cells were fixed in 1% formaldehyde for 15 min, harvested in buffer A (0.25% Triton X-100, 10 mM EDTA, 0.5 mM EGTA, 10 mM HEPES [pH 6.5]), and then resuspended in buffer B (200 mM NaCl, 1 mM EDTA, 0.5 mM EGTA, 10 mM HEPES [pH 6.5]). Cells were then lysed in lysis buffer (1% SDS, 10 mM EDTA, 50 mM Tris-HCl [pH 8.1] with proteinase inhibitors). Chromatin sonication was performed three times for 40 s at setting 5.0 using a Branson 250 sonicator with a microtip. Fragmented chromatin was immunoprecipitated with STAT3 antibodies (SC-482, SC-483; Santacruz Technology, CA, and S21320; Transduction Laboratories, Lexington, KY) for 4 hrs. After reversal of the cross-links and DNA precipitation, enriched DNA was analyzed by PCR amplification with primers that flank the predicted STAT3 TFBSs (Table S2).

## Supporting Information

Figure S1 PWM similarity clustering. (A) Total 565 vertebrate TRANSFAC PWMs were clustered by pair-wise similarity comparison with the Kullback-Leibler divergence. The number of PWM clusters at different similarity P-value cut-offs is plotted. (B) PWM cluster at 10–7 P-value of similarity was represented by Cytoscape [73]
(1.55 MB TIF)

Figure S2 Quality scores of STAT-related PWMs and clustered STAT-related PWMs in the known STAT3 TFBSs. (A) Histogram of PWM quality score for all 565 vertebrate PWMs derived from TRANSFAC ver. 9.4. (B) Number of STAT3 binding sites detected by combined PWMs. Forty STAT3 TFBSs [35] were used as reference dataset. (C) PWM quality score of STAT-related PWMs.
(0.96 MB TIF)

Figure S3 STAT3 TFBS prediction using MATCH and MotifLocator. Curves for the changes of the number of true positive TFBSs detected using MotifLocator (A) or MATCH (B) in the reference set of 22 STAT3 target genes. PWM: V$STAT3_01, V$STAT1_01, or combined PWMs of V$STAT3_01 and V$STAT1_01 (All).
(0.67 MB TIF)

Figure S4 Comparison of the TFBS prediction programs using the genome-wide STAT3 binding. Curves for the changes of the number of true positive TFBSs detected using MATCH (A) or MotifLocator (B) in the genome-wide STAT3 ChIP-Seq dataset.
(0.75 MB TIF)

Figure S5 Estimation of MCS confidence scores. The graph displays confidence scores (dotted line) and predicted numbers of conserved TFBSs (solid line) at each MCS cut-off value.
(0.48 MB TIF)

Figure S6 Genome-wide distribution of predicted STAT3 TFBSs. Using 2.5-kb promoter sequences of all annotated human reference genes, predicted STAT3 TFBSs with STAT-Scanner (blue line at top, P-value <0.1) or STAT-Finder (blue line at bottom, posterior probability >0.5) were plotted. The red line (random) shows the distribution of predicted TFBSs in the randomly permutated promoter sequences.
(0.80 MB TIF)

Figure S7 Experimental validation of STAT3 binding to the novel STAT3 TFBS. The affinity score (top, STAT-Scanner) and posterior probability (middle, STAT-Finder) of the predicted STAT3 TFBS are plotted in the sliding windows for a 2.5-kb promoter region across the ATF3 (A), DUSP5 (C), SERPINE1 (E), NP (G), SLC2A3 (I), and CCL2 (K) genomic loci. The closed square at bottom indicates predicted STAT3 TFBS with posterior probability >0.5. (B, D, F, H, J, L) ChIP analysis with an anti-STAT3 antibody.
(7.45 MB TIF)

Figure S8 Performance comparison of the comparative alignment tools for the STAT3 target genes identified in this study.
(0.36 MB TIF)

Table S1 Lists of the reference set for known STAT3 TFBSs.
(0.17 MB DOC)

Table S2 The information for primer sets used in ChIP experiment.
(0.04 MB DOC)
