# Supplementary figures and images for: Prediction and Experimental Validation of Novel STAT3 Target Genes in Human Cancer Cells
Source: PLoS One. 2009 Sep 4;4(9):e6911. doi: 10.1371/journal.pone.0006911 (PMC2731854; doi:10.1371/journal.pone.0006911)

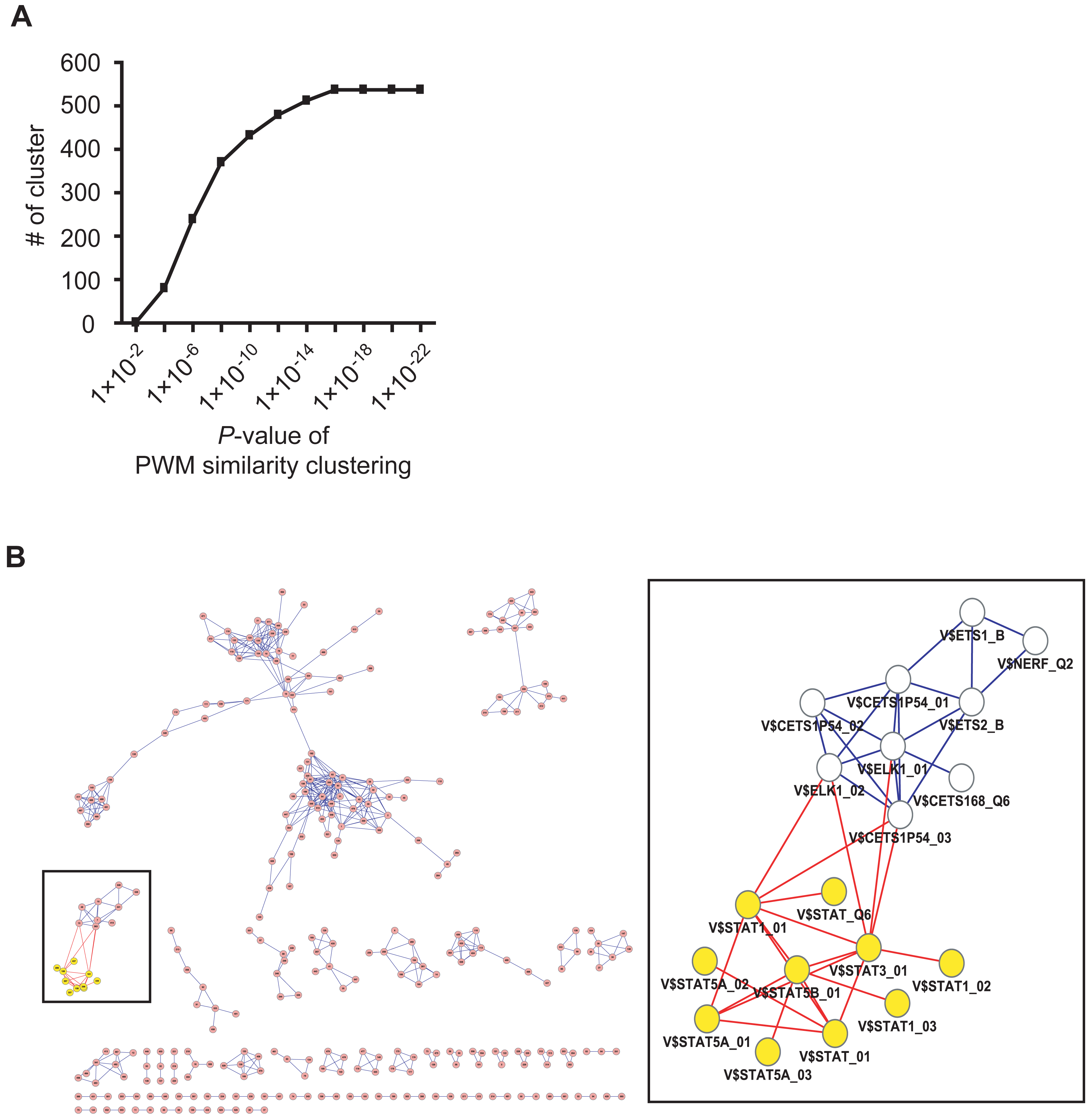

Supplement: Figure S1 — PWM similarity clustering. (A) Total 565 vertebrate TRANSFAC PWMs were clustered by pair-wise similarity comparison with the Kullback-Leibler divergence. The number of PWM clusters at different similarity P-value cut-offs is plotted. (B) PWM cluster at 10–7 P-value of similarity was represented by Cytoscape [73] (1.55 MB TIF) [file pone.0006911.s001.tif]

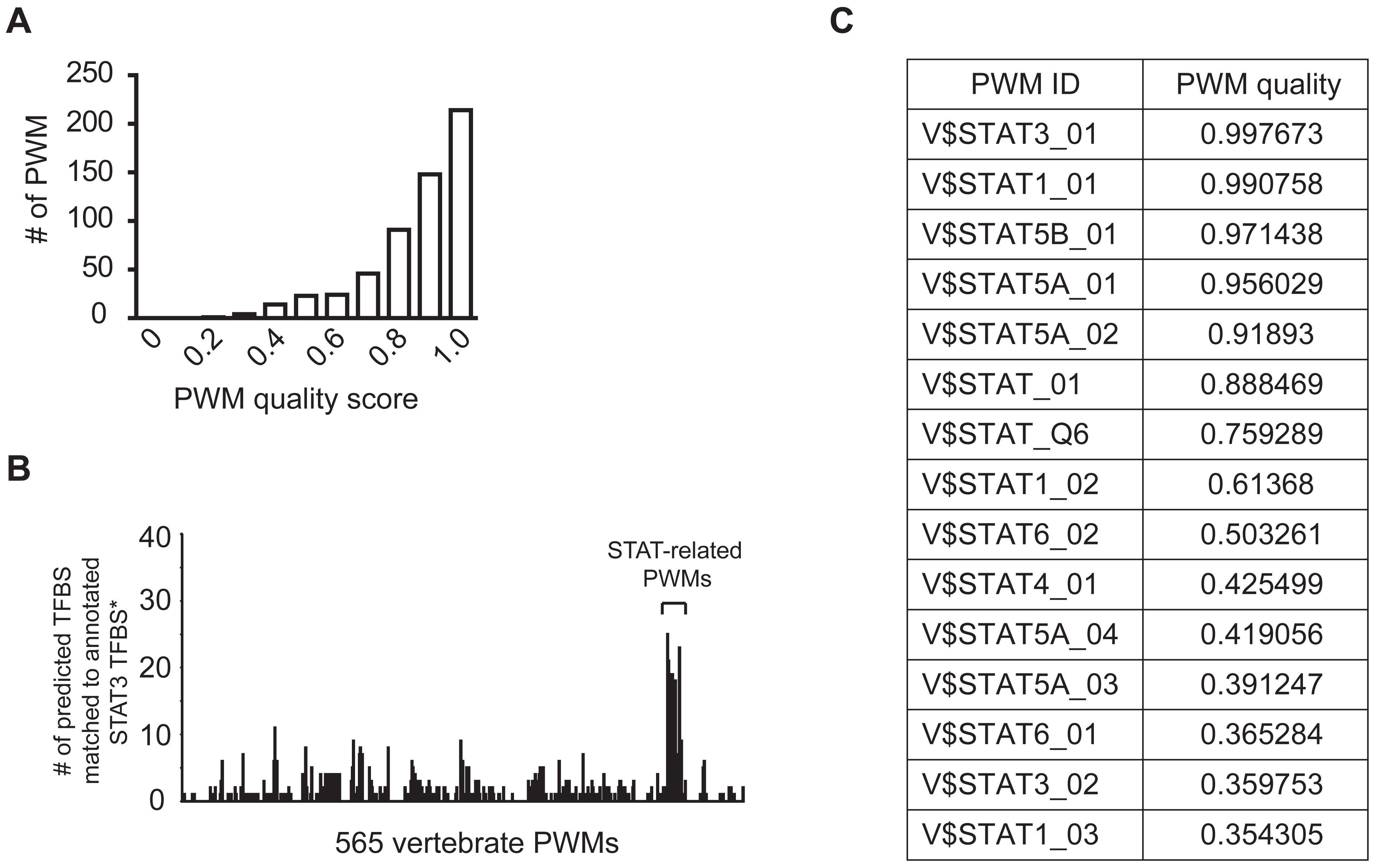

Supplement: Figure S2 — Quality scores of STAT-related PWMs and clustered STAT-related PWMs in the known STAT3 TFBSs. (A) Histogram of PWM quality score for all 565 vertebrate PWMs derived from TRANSFAC ver. 9.4. (B) Number of STAT3 binding sites detected by combined PWMs. Forty STAT3 TFBSs [35] were used as reference dataset. (C) PWM quality score of STAT-related PWMs. (0.96 MB TIF) [file pone.0006911.s002.tif]

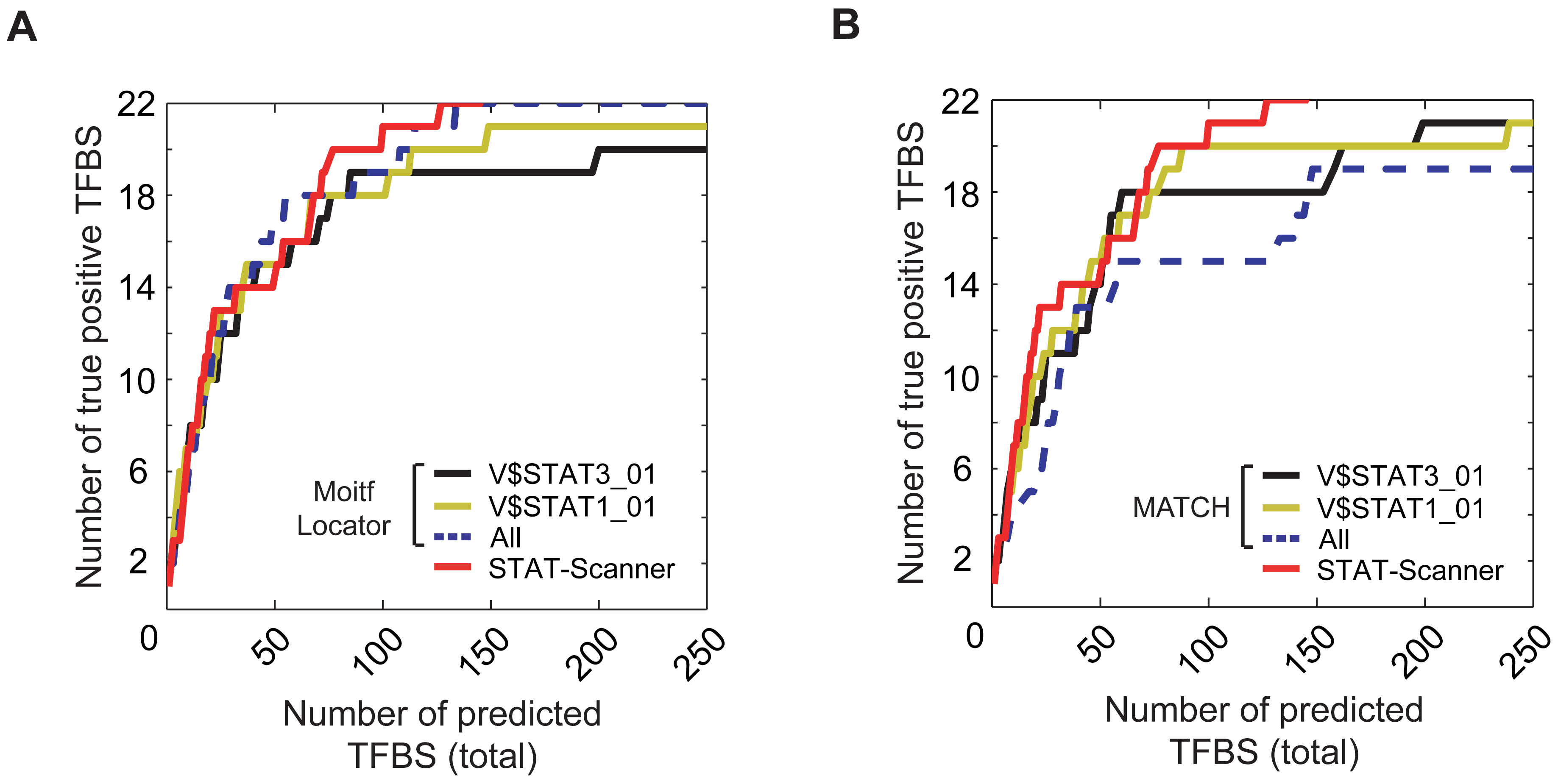

Supplement: Figure S3 — STAT3 TFBS prediction using MATCH and MotifLocator. Curves for the changes of the number of true positive TFBSs detected using MotifLocator (A) or MATCH (B) in the reference set of 22 STAT3 target genes. PWM: V$STAT3_01, V$STAT1_01, or combined PWMs of V$STAT3_01 and V$STAT1_01 (All). (0.67 MB TIF) [file pone.0006911.s003.tif]

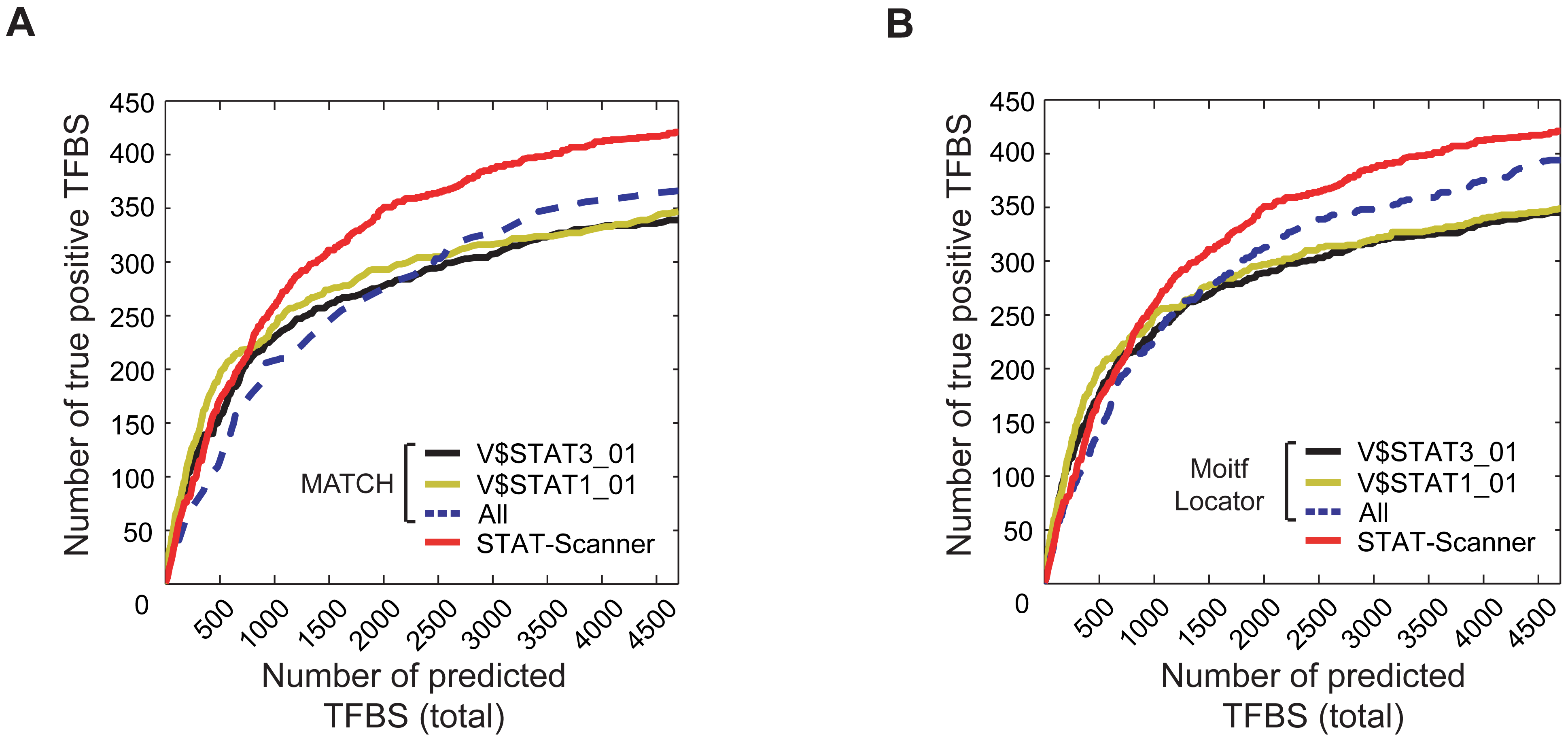

Supplement: Figure S4 — Comparison of the TFBS prediction programs using the genome-wide STAT3 binding. Curves for the changes of the number of true positive TFBSs detected using MATCH (A) or MotifLocator (B) in the genome-wide STAT3 ChIP-Seq dataset. (0.75 MB TIF) [file pone.0006911.s004.tif]

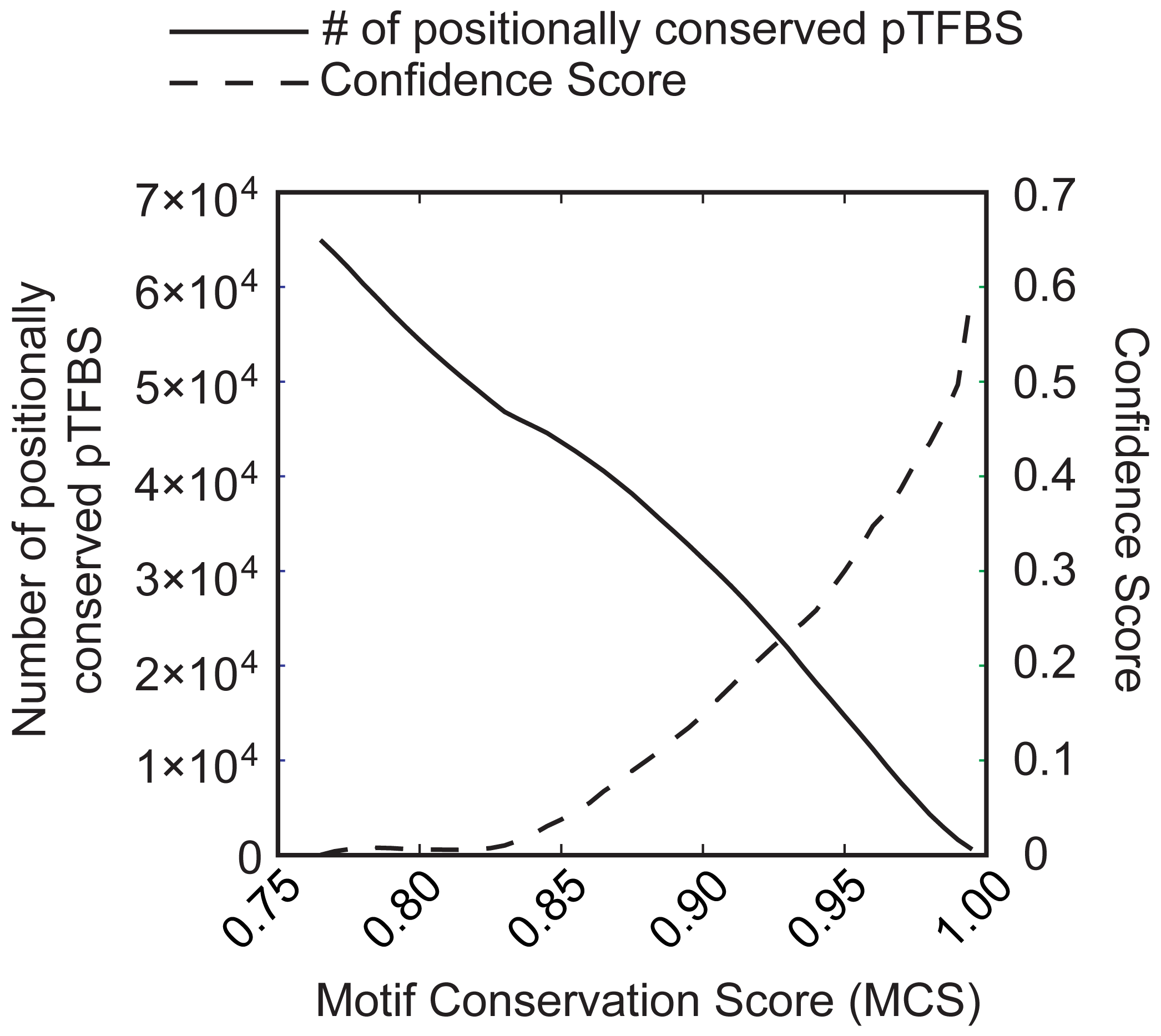

Supplement: Figure S5 — Estimation of MCS confidence scores. The graph displays confidence scores (dotted line) and predicted numbers of conserved TFBSs (solid line) at each MCS cut-off value. (0.48 MB TIF) [file pone.0006911.s005.tif]

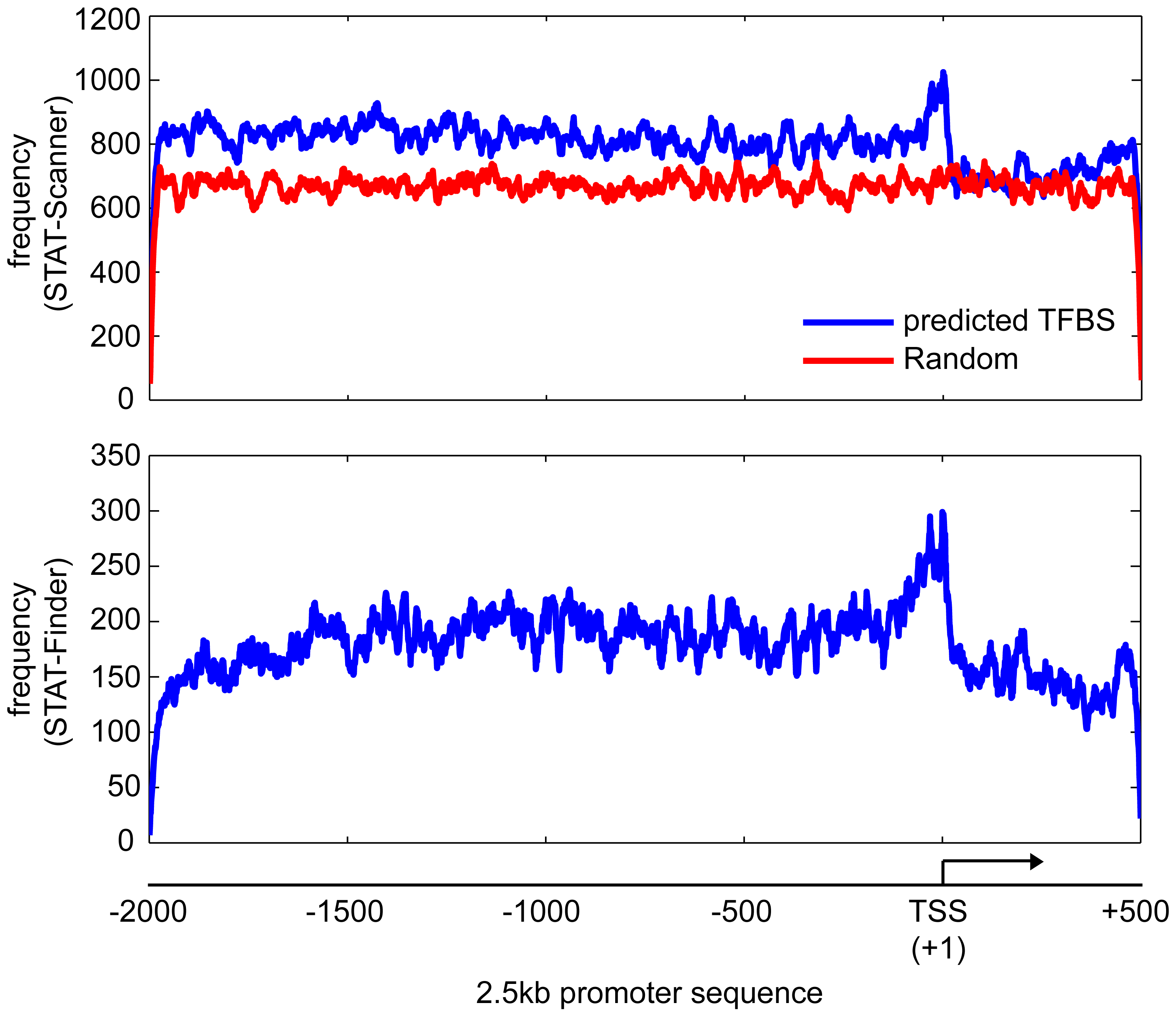

Supplement: Figure S6 — Genome-wide distribution of predicted STAT3 TFBSs. Using 2.5-kb promoter sequences of all annotated human reference genes, predicted STAT3 TFBSs with STAT-Scanner (blue line at top, P-value <0.1) or STAT-Finder (blue line at bottom, posterior probability >0.5) were plotted. The red line (random) shows the distribution of predicted TFBSs in the randomly permutated promoter sequences. (0.80 MB TIF) [file pone.0006911.s006.tif]

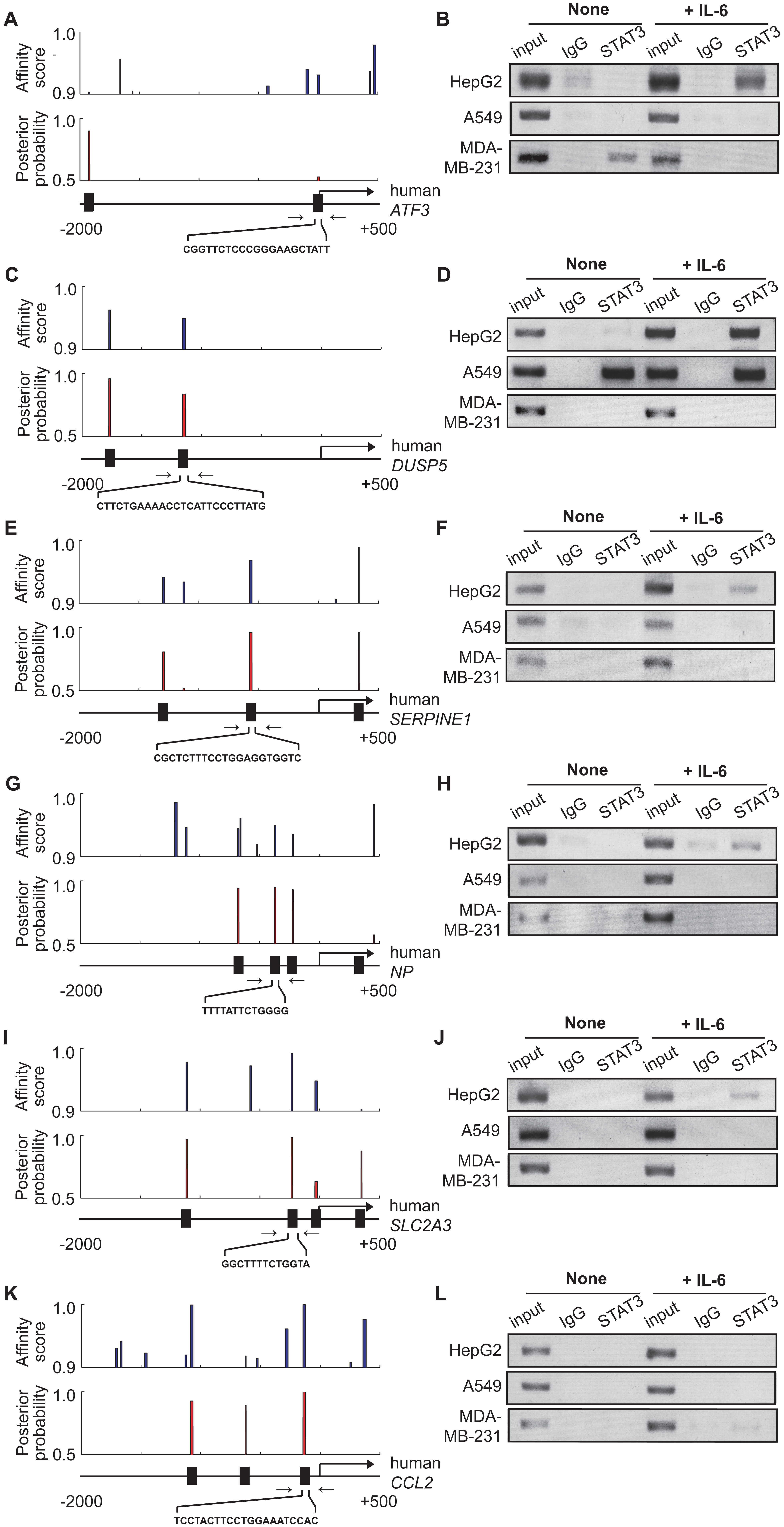

Supplement: Figure S7 — Experimental validation of STAT3 binding to the novel STAT3 TFBS. The affinity score (top, STAT-Scanner) and posterior probability (middle, STAT-Finder) of the predicted STAT3 TFBS are plotted in the sliding windows for a 2.5-kb promoter region across the ATF3 (A), DUSP5 (C), SERPINE1 (E), NP (G), SLC2A3 (I), and CCL2 (K) genomic loci. The closed square at bottom indicates predicted STAT3 TFBS with posterior probability >0.5. (B, D, F, H, J, L) ChIP analysis with an anti-STAT3 antibody. (7.45 MB TIF) [file pone.0006911.s007.tif]

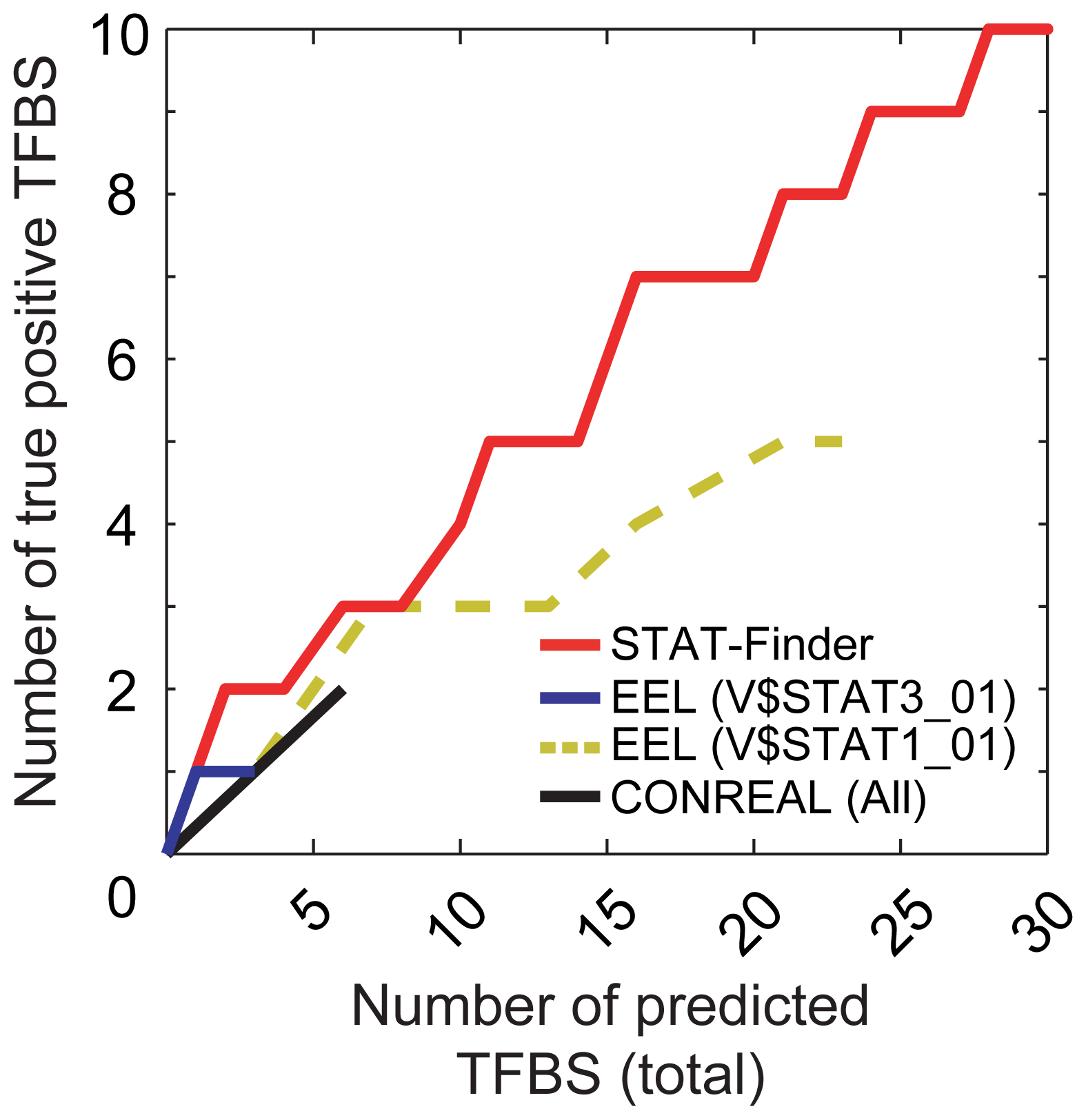

Supplement: Figure S8 — Performance comparison of the comparative alignment tools for the STAT3 target genes identified in this study. (0.36 MB TIF) [file pone.0006911.s008.tif]
