# Supplementary material for: Prediction and Experimental Validation of Novel STAT3 Target Genes in Human Cancer Cells
Source: PLoS One. 2009 Sep 4;4(9):e6911. doi: 10.1371/journal.pone.0006911 (PMC2731854; doi:10.1371/journal.pone.0006911)
Supplement: Table S1 — Lists of the reference set for known STAT3 TFBSs. (0.17 MB DOC) [file pone.0006911.s009.doc]

| **Gene** | **^a^Species** | **^b^Position /**  **UCSC browser** | ***STAT3 TFBS Sequences*** | **^c^STAT3 TFBS Positions** | **Ref** |
| --- | --- | --- | --- | --- | --- |
| *A2m* | R | chr4:158,101,711-158,104,210 | TTCTGGGAA | -193 ~ -185 | [1] |
| *AGT* | H | chr1:228,916,065-228,918,564 | TTCTGGGAA | -203 ~ -195 | [2] |
| *CCND1* | H | chr11:69,163,054-69,165,553 | TTCCAGCAA | -936 ~ -928 | [3] |
| *CDKN1A* | H | chr6:36,752,465-36,754,964 | CTCCTTCCCGGAAGCA | -682 ~ -667 | [4] |
| *FCGR1A* | H | chr1:148,018,912-148,021,411 | GTATTTCCCAGAAAAGCAAC | -134 ~ -115 | [5] |
| *FOS* | H | chr14:74,813,284-74,815,783 | AGTTCCCGTCAAT | -349 ~ -337 | [6] |
| *Fos* | M | chr12:86,812,851-86,815,350 | CTGTTCCCGTCAATCCCTCCC | -357 ~ -337 | [7] |
| *HGF* | H | chr7:81,236,889-81,239,388 | AGTTTCTTACCGTAAGAGGG | -16 ~ +3 | [8] |
| *IL18R1* | H | chr2:102,343,529-102,346,028 | TTCTGAGAA | -154 ~ -146 | [9] |
| *IL6ST* | H | chr5:55,326,021-55,328,520 | GCGTTACGGGAATCG | -247 ~ -233 | [10] |
| *IRF1* | H | chr5:131,853,827-131,856,326 | CTGATTTCCCCGAAATGAC | -166 ~ -144 | [11] |
| *MCL1* | H | chr1:148,818,261-148,820,760 | CCTTTCCCCTTTTATGGGAATACTTTTTTTAAAAA | -52 ~ -18 | [12] |
| *MMP2* | H | chr16:54,068,589-54,071,088 | CTGCTCTATTTCCCAAGGTGTATC | -607 ~ -584 | [13] |
| *Mt1* | M | chr8:96,701,127-96,703,626 | GAGTTCTCGTAAACTC | -288 ~ -273 | [14] |
| *MUC1* | H | chr1:153,428,825-153,431,324 | GGCTATTCCGGGAAGTGGT | -513 ~ -495 | [15] |
| *PIM1* | H | chr6:37,243,964-37,246,463 | ACACACATCCCTTCCCAGAAATCAGGATTC | -995 ~ -966 | [16] |
| *Pomc* | M | chr12:3,952,952-3,955,451 | TAGTGATATTTACCTCCAAATGCCAGGAAGGC | -381 ~ -350 | [17] |
| *PRF1* | H | chr10:72,032,038-72,034,537 | TGGGGCCAGATTCCGAGAAGACAGCAT | -1065 ~ -1039 | [18] |
| *SERPINA3* | H | chr14:94,146,468-94,148,967 | CCGTATTACCAGAAATTATC | -116 ~ -97 | [19] |
| *Socs3* | M | chr11:117,829,977-117,832,476 | CAGTTCCAGGAATCGGGGGGC | -87 ~ -67 | [20] |
| *Trh* | M | chr6:92,194,143-92,196,642 | CCACCAGGTTTCCGGAAA | -138 ~ -121 | [21] |
| *VEGFA* | H | chr6:43,843,931-43,846,430 | ATCCCTGGACACTTCCCAAAGGAC | -866 ~ -843 | [22] |

^a^H, human; M, mouse; R, rat

^b^Location of the 2.5kb promoter sequence obtained from the UCSC Genome browser (Human: Mar. 2006 Assembly, Mouse: July 2007 Assembly, Rat: Nov. 2004 Assembly)

^c^Start and end position of the known STAT3 TFBS in the promoter

## References

1. Zhang X, Darnell JE, Jr. (2001) Functional importance of Stat3 tetramerization in activation of the alpha 2-macroglobulin gene. J Biol Chem 276: 33576-33581.

2. Sherman CT, Brasier AR (2001) Role of signal transducers and activators of transcription 1 and -3 in inducible regulation of the human angiotensinogen gene by interleukin-6. Mol Endocrinol 15: 441-457.

3. Lo HW, Hsu SC, Ali-Seyed M, Gunduz M, Xia W, et al. (2005) Nuclear interaction of EGFR and STAT3 in the activation of the iNOS/NO pathway. Cancer Cell 7: 575-589.

4. Chen B, He L, Savell VH, Jenkins JJ, Parham DM (2000) Inhibition of the interferon-gamma/signal transducers and activators of transcription (STAT) pathway by hypermethylation at a STAT-binding site in the p21WAF1 promoter region. Cancer Res 60: 3290-3298.

5. Wehinger J, Gouilleux F, Groner B, Finke J, Mertelsmann R, et al. (1996) IL-10 induces DNA binding activity of three STAT proteins (Stat1, Stat3, and Stat5) and their distinct combinatorial assembly in the promoters of selected genes. FEBS Lett 394: 365-370.

6. Yang E, Lerner L, Besser D, Darnell JE, Jr. (2003) Independent and cooperative activation of chromosomal c-fos promoter by STAT3. J Biol Chem 278: 15794-15799.

7. Rajotte D, Sadowski HB, Haman A, Gopalbhai K, Meloche S, et al. (1996) Contribution of both STAT and SRF/TCF to c-fos promoter activation by granulocyte-macrophage colony-stimulating factor. Blood 88: 2906-2916.

8. Tomida M, Saito T (2004) The human hepatocyte growth factor (HGF) gene is transcriptionally activated by leukemia inhibitory factor through the Stat binding element. Oncogene 23: 679-686.

9. Sahar S, Dwarakanath RS, Reddy MA, Lanting L, Todorov I, et al. (2005) Angiotensin II enhances interleukin-18 mediated inflammatory gene expression in vascular smooth muscle cells: a novel cross-talk in the pathogenesis of atherosclerosis. Circ Res 96: 1064-1071.

10. Funamoto M, Hishinuma S, Fujio Y, Matsuda Y, Kunisada K, et al. (2000) Isolation and characterization of the murine cardiotrophin-1 gene: expression and norepinephrine-induced transcriptional activation. J Mol Cell Cardiol 32: 1275-1284.

11. Andersen P, Pedersen MW, Woetmann A, Villingshoj M, Stockhausen MT, et al. (2008) EGFR induces expression of IRF-1 via STAT1 and STAT3 activation leading to growth arrest of human cancer cells. Int J Cancer 122: 342-349.

12. Isomoto H, Kobayashi S, Werneburg NW, Bronk SF, Guicciardi ME, et al. (2005) Interleukin 6 upregulates myeloid cell leukemia-1 expression through a STAT3 pathway in cholangiocarcinoma cells. Hepatology 42: 1329-1338.

13. Xie TX, Wei D, Liu M, Gao AC, Ali-Osman F, et al. (2004) Stat3 activation regulates the expression of matrix metalloproteinase-2 and tumor invasion and metastasis. Oncogene 23: 3550-3560.

14. Lee DK, Carrasco J, Hidalgo J, Andrews GK (1999) Identification of a signal transducer and activator of transcription (STAT) binding site in the mouse metallothionein-I promoter involved in interleukin-6-induced gene expression. Biochem J 337 ( Pt 1): 59-65.

15. Gaemers IC, Vos HL, Volders HH, van der Valk SW, Hilkens J (2001) A stat-responsive element in the promoter of the episialin/MUC1 gene is involved in its overexpression in carcinoma cells. J Biol Chem 276: 6191-6199.

16. Matikainen S, Sareneva T, Ronni T, Lehtonen A, Koskinen PJ, et al. (1999) Interferon-alpha activates multiple STAT proteins and upregulates proliferation-associated IL-2Ralpha, c-myc, and pim-1 genes in human T cells. Blood 93: 1980-1991.

17. Mynard V, Latchoumanin O, Guignat L, Devin-Leclerc J, Bertagna X, et al. (2004) Synergistic signaling by corticotropin-releasing hormone and leukemia inhibitory factor bridged by phosphorylated 3',5'-cyclic adenosine monophosphate response element binding protein at the Nur response element (NurRE)-signal transducers and activators of transcription (STAT) element of the proopiomelanocortin promoter. Mol Endocrinol 18: 2997-3010.

18. Yu CR, Ortaldo JR, Curiel RE, Young HA, Anderson SK, et al. (1999) Role of a STAT binding site in the regulation of the human perforin promoter. J Immunol 162: 2785-2790.

19. Kordula T, Rydel RE, Brigham EF, Horn F, Heinrich PC, et al. (1998) Oncostatin M and the interleukin-6 and soluble interleukin-6 receptor complex regulate alpha1-antichymotrypsin expression in human cortical astrocytes. J Biol Chem 273: 4112-4118.

20. Auernhammer CJ, Bousquet C, Melmed S (1999) Autoregulation of pituitary corticotroph SOCS-3 expression: characterization of the murine SOCS-3 promoter. Proc Natl Acad Sci U S A 96: 6964-6969.

21. Harris M, Aschkenasi C, Elias CF, Chandrankunnel A, Nillni EA, et al. (2001) Transcriptional regulation of the thyrotropin-releasing hormone gene by leptin and melanocortin signaling. J Clin Invest 107: 111-120.

22. Wei D, Le X, Zheng L, Wang L, Frey JA, et al. (2003) Stat3 activation regulates the expression of vascular endothelial growth factor and human pancreatic cancer angiogenesis and metastasis. Oncogene 22: 319-329.
