# Supplementary material for: Prediction and Experimental Validation of Novel STAT3 Target Genes in Human Cancer Cells
Source: PLoS One. 2009 Sep 4;4(9):e6911. doi: 10.1371/journal.pone.0006911 (PMC2731854; doi:10.1371/journal.pone.0006911)
Supplement: Table S2 — The information for primer sets used in ChIP experiment. (0.04 MB DOC) [file pone.0006911.s010.doc]

| Gene symbol | Remark for ChIP experiment | Forward primer sequence | Reverse primer sequence | PCR product length (bp) |
| --- | --- | --- | --- | --- |
| *JUNB* | positive control | 5’-CCAGTGGACTCCAGGGAAATC-3’ | 5’-GCGCTAGTCAGCCACGGAAG-3’ | 278 |
| *MYC* | positive control | 5’-CGAGGGTCTGGACGGCTG-3’ | 5’-GCTCGCCCGGCTCTTC-3’ | 258 |
| *NNMT* | positive control | 5’-CAGAGGCAGGATGTGCTGC-3’ | 5’-TCACTCCCCGCTAAAGCATTC-3’ | 304 |
| *AKAP12* | putative target | 5’-GCTGACGGCAAAGGAACAG-3’ | 5’-ACGCAGGCCCACGCTC-3’ | 225 |
| *AKAP12* | non-conserved TFBS (internal negative control) | 5’-CCTACTCCTCCCCCTCCTG-3’ | 5’-CACGAAGGCCAATTTCTCTG-3’ | 244 |
| *ATF3* | putative target | 5’-TCCGGTCCTGATATGGAGAGAG-3’ | 5’-TCGTTTACTCCGTGTTGCCAG-3’ | 241 |
| *CCL2* | putative target | 5’-CCTCCCCATTTGCTCATTT-3’ | 5’-CTGCTGTCTCTGCCTCTTATTG-3’ | 213 |
| *DUSP5* | putative target | 5’-CGTCTTCACCAAGACCCCAC-3’ | 5’-CTGGCTGGGACTGCTTGATG-3’ | 222 |
| *HIC2* | putative target | 5’-CGGCTATTTATTTTTCCAGGTG-3’ | 5’-GTCGCCCAAGTCCAAGAG-3’ | 208 |
| *HIC2* | non-conserved TFBS (internal negative control) | 5’-TGAGGTGATTTATGTGCTTCCA-3’ | 5’-CAGGAGAATGGCGTGAACC-3’ | 244 |
| *NP* | putative target | 5’-AGCTTCCCTTGTCCAGGGAG-3’ | 5’-TCATCTCGGTACCCATTCCAG-3’ | 240 |
| *SERPINE1* | putative target | 5’-ATGGCAGGGATGAGGGAAAG-3’ | 5’-GCTCACGTTGCCTGCTTTTC-3’ | 261 |
| *SLC2A3* | putative target | 5’-TAGCACCCACTTGACTTCGTT-3’ | 5’-CACATCCTTCCACTCTCTCCA-3’ | 245 |
| *THBS1* | putative target | 5’-ACGGGCCCAGTCTCTAGTATCC-3’ | 5’-GGCGCGCAACTTTCCAG-3’ | 290 |
| *THBS1* | non-conserved TFBS (internal negative control) | 5’-ACCTATGATTTGCGGGACAA-3’ | 5’-CTTTTCTTGCGTGGGAGTGT-3’ | 234 |
| *DNMT* | Negative control for ChIP against STAT3 | 5’-AGGTGGGTGGATCACTTGAG-3’ | 5’-GTGGAACAGCCAACAATCCT-3’ | 299 |
| *HEPCIDIN* | Negative control for ChIP against STAT3 | 5’-TCTCTGCCTTCAGTGCCTTT-3’ | 5’-CACTTCTGCACCAACTCAGC-3’ | 208 |
